# G protein-coupled receptor 37 is a negative regulator of oligodendrocyte differentiation and myelination

**DOI:** 10.1038/ncomms10884

**Published:** 2016-03-10

**Authors:** Hyun-Jeong Yang, Anna Vainshtein, Galia Maik-Rachline, Elior Peles

**Affiliations:** 1 Department of Molecular Cell Biology, Weizmann Institute of Science, Rehovot 76100, Israel

## Abstract

While the formation of myelin by oligodendrocytes is critical for the function of the central nervous system, the molecular mechanism controlling oligodendrocyte differentiation remains largely unknown. Here we identify G protein-coupled receptor 37 (GPR37) as an inhibitor of late-stage oligodendrocyte differentiation and myelination. GPR37 is enriched in oligodendrocytes and its expression increases during their differentiation into myelin forming cells. Genetic deletion of *Gpr37* does not affect the number of oligodendrocyte precursor cells, but results in precocious oligodendrocyte differentiation and hypermyelination. The inhibition of oligodendrocyte differentiation by GPR37 is mediated by suppression of an exchange protein activated by cAMP (EPAC)-dependent activation of Raf-MAPK-ERK1/2 module and nuclear translocation of ERK1/2. Our data suggest that GPR37 regulates central nervous system myelination by controlling the transition from early-differentiated to mature oligodendrocytes.

## Abstract (web summary)

The molecular mechanism controlling oligodendrocyte differentiation is not fully understood. Here the authors show that G protein coupled receptor 37 acts as a negative regulator of CNS myelination, and this effect is mediated by suppression of ERK signalling.

## Graphical abstract

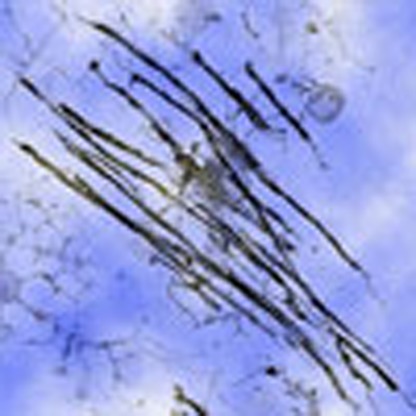

The myelin membrane, produced by Schwann cells in the peripheral nervous system (PNS) and oligodendrocytes in the central nervous system (CNS) enables energy-efficient saltatory conduction, and provides essential trophic support to maintain axonal integrity and survival^1^. Myelination is a late developmental process that continues to be remodelled throughout life, suggesting that it contributes to nervous system plasticity^2^,^3^. Destruction of myelin in the CNS not only leads to devastating white matter diseases such as leukodystrophies^4^ and multiple sclerosis but also is associated with psychiatric disorders^5^, and neurodegenerative diseases. Hence, understanding the mechanisms underlying oligodendrocyte development and myelination, as well as their maintenance and ability to remyelinate after damage, is of great clinical interest^6^.

During development, oligodendrocyte precursor cells (OPCs) differentiate into post-mitotic pre-myelinating oligodendrocytes, which later on continue to myelinate. Several signalling pathways control the intricate balance between OPC proliferation and differentiation^7^,^8^. Myelination in the CNS is regulated by both inhibitory (for example, PSA-NCAM (ref. 9), WNT (ref. 10), LINGO (ref. 11), GPR17 (ref. 12) and Notch-1 (ref. 13)), and stimulatory (for example, laminin-α2 (ref. 14), BDNF (ref. 15) and FGF receptor 2 (ref. 16)) signals. Nevertheless, it is not clear whether once OPCs exit the cell cycle and begin to differentiate, additional extrinsic signals are required for the progression from pre-myelinating to myelinating oligodendrocytes^1^,^17^.

Members of the G protein-coupled receptors (GPCRs) superfamily are emerging as important regulators of myelination. For example, in the PNS, the initiation of myelination requires the presence of the adhesion-type GPR126 in Schwann cells^18^,^19^. PNS myelin formation and maintenance are also modulated by GPR44, which is activated by prostaglandin D2 (ref. 20). In the CNS, OPCs proliferation and early differentiation are regulated by the adhesion-type GPR56 protein^21^,^22^ and GPR17 (ref. 12), respectively. Mutations in GPR56 cause bilateral frontoparietal polymicrogyria disease, which is also characterized by white matter reduction^23^. In addition, several other GPCRs were implicated in remyelination, but their roles in myelination during development remains to be determined^24^,^25^,^26^.

We have previously combined microarray analysis with genetic ablation of oligodendrocytes in mice to identify novel signalling pathways involved in late developmental stages of CNS myelination^27^,^28^. This approach resulted in the identification of GPCR 37 (*Gpr37*) as an oligodendrocyte-enriched gene. GPR37 shares significant homology with the receptors of endothelin and bombesin peptides^29^. As revealed by cell-specific gene expression analysis^30^ GPR37 is predominantly found in pre-myelinating and myelinating oligodendrocytes, but not in OPCs. It is also present in certain neuronal subsets, such as dopaminergic neurons in the substantia nigra^31^. GPR37 is also known as parkin-associated endothelin B-like receptor (PAEL-R), which was identified as one of the substrates of the E3 ubiquitin ligase parkin^31^. Although expressed mostly in white matter, the function of GPR37 in myelination has not yet been addressed. Here we show that GPR37 is a negative regulator of oligodendrocytes differentiation and myelination and that its function is mediated by suppression of an Epac-dependent activation of MAPK cascade and nuclear translocation of ERK1/2. Our data also suggest that the differentiation of oligodendrocytes to myelinating cells is controlled by the sequential action of inhibitory GPCRs.

## Results

### *Gpr37* is an oligodendrocyte-enriched gene

**Figure 1:**
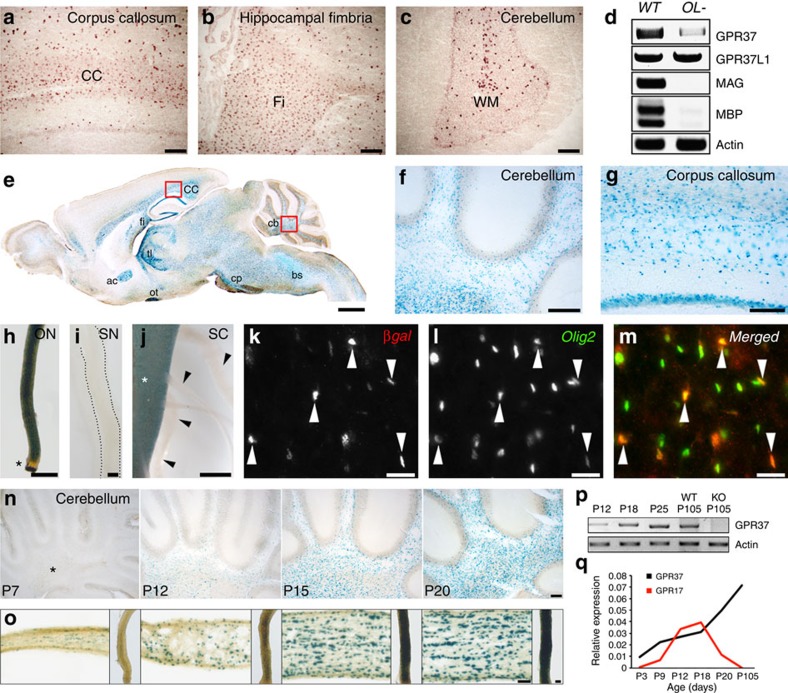
Gpr37 is enriched in myelinating glia in the CNS. (**a**–**c**) *In situ* hybridization. Sagittal-sections of adult rat brain were hybridized to GPR37 antisense probe. GPR37 mRNA is detected in white matter areas: corpus callosum (**a**; CC), hippocampal fimbria (**b**; Fi) and the cerebellar white matter tracks (**c**; WM). (**d**) RT–PCR of brain RNA isolated from P12 wild-type (*WT*) and oligodendrocyte-ablated mice (*OL-*). MAG and MBP were used to monitor genes that are expressed specifically in oligodendrocytes, while actin was used as a control for ubiquitously expressed genes. (**e**) β-gal staining in a sagittal brain section of adult mice carrying a LacZ allele in the *Gpr37* locus: cerebellum (cb), brainstem (bs), cerebral peduncle (cp), corpus callosum (cc), hippocampal fimbria (fi), thalamus (tl), anterior commissure (ac) and optic tract (ot). (**f**,**g**) Higher magnification of the boxed areas in **e**. (**h**–**j**) Lac Z activity monitored in whole mount preparations of optic nerve (ON), sciatic nerve (SN), and spinal cord (SC) as indicated. Representative pictures are from P70 heterozygous mice (**h**) and P20 (**i**) or P15 (**j**) homozygous mice. GPR37 is absent in the unmyelinated part of the optic nerve (asterisk in **h**), as well as in sciatic nerve (**i**) and spinal nerve roots (arrowheads in **j**) emanating from the spinal cord (asterisk in **j**). (**k**–**m**) Immunolabelling of P12 mice caudate putamen using antibodies to βgal and Olig2. (**n**,**o**) Expression of LacZ in the cerebellum (**n**), and optic nerve (**o**) isolated from P7, P12, P15 and P20 heterozygous mice. Asterisk mark the location of the white matter. (**p**) RT–PCR analysis of GPR37 mRNA expression in mouse brain at the indicated postnatal days. Primers to actin were used as control. The expression of GPR37 at P105 was compared with *Gpr37*^*−/−*^ (KO) mice. (**q**) Relative mRNA levels of GPR37 and GPR17 determined by real-time PCR analysis of whole brain RNA. Scale bars, (**a**–**c**) 100 μm; (**e**) 1 mm; (**f**,**g**) 200 μm; (**h**–**j**) 50 μm; (**k**–**m**) 20 μm; (**n**–**o**) 100 μm.

To examine the expression of GPR37, we performed *in situ* hybridization of adult rat brains (Fig. 1a–c). GPR37 expression was mainly detected in white matter areas, such as the caudate putamen, corpus callosum, hippocampal fimbria and cerebellum. To distinguish between the expression of GPR37 in oligodendrocytes versus other cell types, we performed PCR with reverse transcription (RT–PCR) analysis of brain mRNA isolated from wild-type and R26;lacZbpA(flox)DTA mice, in which oligodendrocytes were eliminated using a binary genetic system^27^,^28^. In support of the *in situ* results, the expression of GPR37 was markedly reduced after genetic depletion of oligodendrocytes (Fig. 1d). In contrast, the amount of the related receptor GPR37L1 was unchanged in the absence of oligodendrocytes, consistent with its expression in astrocytes^30^. To further characterize the spatial and temporal expression of GPR37, we made use of the B6.129P2-*Gpr37*^*tm1Dgen*^/J mouse line (hereafter referred to as *Gpr37*^*−/−*^), which contains a bacterial LacZ reporter gene in the *Gpr37* locus^32^. β-galactosidase (βgal) staining of brain slices revealed a strong expression of GPR37 in white matter fibre tracts, such as the cerebellum, corpus callosum, anterior commissure, fimbria and cerebral peduncle (Fig. 1e–g). We also noted the expression of GPR37 in hippocampal neurons. A strong signal was detected in the optic nerve (Fig. 1h) and spinal cord (Fig. 1j), but not in the sciatic nerve (Fig. 1i) or the spinal roots (Fig. 1j). Immunolabelling of P12 mouse caudate putamen using antibodies to βgal and Olig2 revealed that Gpr37 was indeed present in Olig2-positive cells (Fig. 1k–m). Notably, the intensity of βgal was inversely correlated with Olig2, which is downregulated during oligodendrocyte differentiation^8^. Developmental analysis of the cerebellum and optic nerves demonstrated that the expression of GPR37 gradually increased as myelination progressed (Fig. 1n,o). RT–PCR and quantitative real-time PCR (qRT-PCR) analysis of mouse brains revealed that GPR37 transcript could be detected already at P3, and steadily increased to adulthood (Fig. 1p). This pattern of expression was distinct from Gpr17, which decreases at P20 (Fig. 1q). Furthermore, immunolabelling of P12 brainstem revealed that the expression of these two GPCRs is mutually exclusive, (that is, only ∼4% of the cells expressing either Gpr37 or Gp17 cells were positive for both GPCRs) (Supplementary Fig. 1). While Gpr17 is mainly detected in OPCs and early-differentiated cells, GPR37 is mainly present in more mature cells that do not express GPR17. These results show that GPR37 is highly expressed in myelinating glia in the CNS, that its expression increased with myelination, and persists in mature myelinating oligodendrocytes in the adult. This conclusion is in line with recent observations demonstrating the expression of GPR37 in human oligodendrocytes^33^.

### *GPR37* deletion accelerates oligodendrocyte differentiation

**Figure 2:**
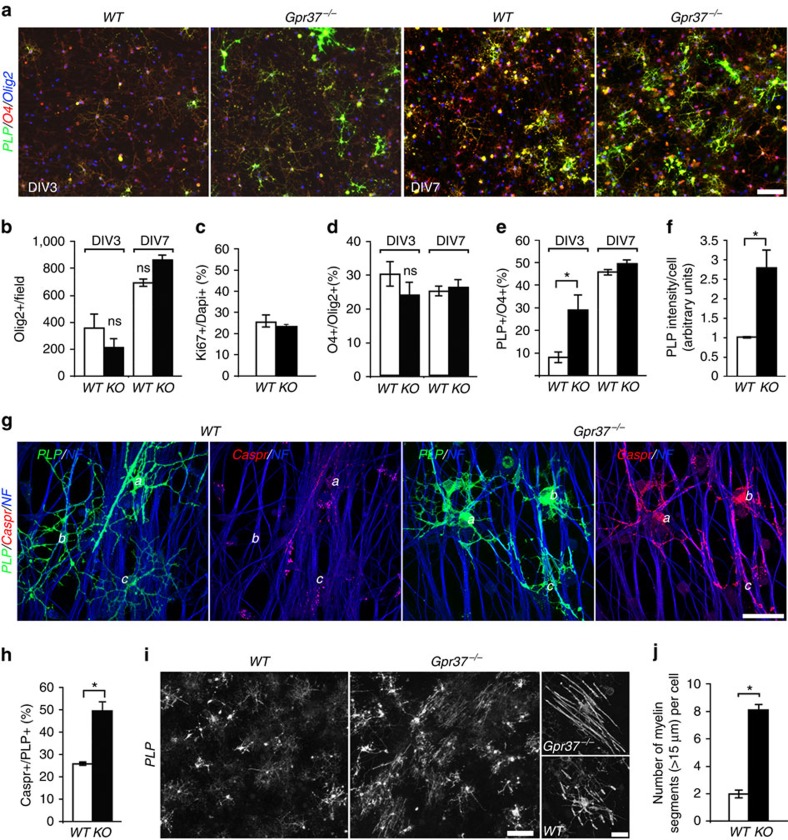
Absence of *Gpr37* results in faster differentiation of oligodendrocytes. (**a**) Immunolabelling of wild-type (WT) or *Gpr37*^*−/−*^ OPCs co-cultured for 3 (DIV3) or 7 (DIV7) days with DRG neurons, using antibodies to Olig2, O4 and PLP. (**b**) The number of Olig2-positive cells (per field of view). (**c**) Percentage of proliferating cells (labelled for ki67) at DIV3 is comparable between WT and *Gpr37*^*−/−*^ cultures. Total 2111 and 2076 cells were investigated for WT and *Gpr37*^*−/−*^, respectively. (**d**) Percentage of O4-positive oligodendrocytes among the total population of Olig2-positive cells (that is, early differentiation). (**e**) Percentage of PLP-positive-oligodendrocytes among the total population of O4-positive cells (that is, late differentiation). The number of cells already expressing PLP at DIV3 is significantly higher in *Gpr37*^*−/−*^ compared with wild-type oligodendrocytes (**e**; *P<0.05, *n*=3 different cultures per each genotype at the indicated time point). (**f**) Relative fluorescence intensity (arbitrary units) of PLP at DIV7 (*P<0.05, *n*=3 cultures per each genotype). (**g**) Immunolabelling of wild-type or *Gpr37*^*−/−*^ OPCs co-cultured for 9 (DIV9) days with DRG neurons, using antibodies to PLP, Caspr, and neurofilament (NF). Three different PLP-positive oligodendrocytes (label: **a**–**c**) are marked in each panel. In contrast to the wild-type co-culture, PLP-positive oligodendrocytes in *Gpr37*^*−/−*^ coculture are associated with intense clustering of Caspr at contact sites along the axons. (**h**) Quantitation of the results showing the per cent of PLP-positive cells that associate with axonal Caspr immunoreactivity (*P<0.05, *n*=3 different cultures per each genotype). (**i**) Advanced myelination by *Gpr37*^*−/−*^ oligodendrocytes. Co-cultures (DIV9) prepared using oligodendrocytes of each genotype together with wild-type mouse DRG neurons labelled with an antibody to PLP. High magnification images are shown on the right. (**j**) The number of PLP-positive myelin segments longer than 15 μm per cell is shown. Bars represent mean±s.e.m. *P<0.001, total of 100 cells each genotype were analysed). Scale bars, (**a**,**i**) 100 μm; (**g**) 40 μm; (**i**) (insets), 20 μm.

During the development, OPCs differentiate along a defined pathway, marked by a typical change in cell morphology and expression of myelin-specific lipids and proteins^34^. To begin evaluating the role GPR37 plays in CNS myelination, we followed the differentiation of wild-type and *Gpr37*^*−/−*^ oligodendrocytes cultured with wild-type dorsal root ganglion (DRG) neurons. Antibodies to Olig2, O4 and proteolipid protein (PLP) were used as early, mid- and late-stage differentiation markers, respectively (Fig. 2a). Three (DIV3) or 7 (DIV7) days after plating, the number of Olig2-positive cells was not significantly different between wild-type and *Gpr37*^*−/−*^ cultures (Fig. 2b), indicating that GPR37 is not required for the generation of OPCs. Accordingly, we found that the absence of GPR37 did not increase cell proliferation, as evaluated by immunolabelling for Ki-67 at DIV3 (Fig. 2c). At both time points, the percentage of Olig2 cells expressing O4 was not significantly different between the two genotypes (Fig. 2d), suggesting that the early differentiation of OPCs was not affected by the deletion of GPR37. In contrast, the number of O4-positive cells already expressing PLP was significantly higher in the *Gpr37*^*−/−*^ cultures at DIV3 (Fig. 2e). At DIV7, although the number of PLP expressing oligodendrocytes was similar in the two cultures (Fig. 2e), the intensity of PLP-immunoreactivity was >2.5-fold higher in the *Gpr37*^*−/−*^ culture (Fig. 2a, right panel, f). Similarly, acute shRNA knockdown of GPR37 in OPC co-cultured with DRG neurons resulted in enhanced oligodendrocyte differentiation (Supplementary Fig. 2).

To further assess the difference between wild-type and *Gpr37*^*−/−*^ cultures, we examined the distribution of the axonal protein Caspr, which is clustered upon oligodendrocyte contact and ensheathment^35^,^36^ (a process referred to as ‘Caspr mirroring', which occurs just before myelination and formation of the paranodal junction). Immunolabelling of DIV9 cultures using antibodies to Caspr and PLP demonstrated that although the number of PLP cells was similar in both genotypes, the number of PLP-positive oligodendrocytes that were associated with intense axonal clustering of Caspr was significantly higher in *Gpr37*^*−/−*^ than in wild-type co-cultures (Fig. 2g,h). Finally, immunolabelling of OPC/DRG neurons co-cultures at DIV9 with an antibody to PLP revealed that at this stage oligodendrocytes lacking GPR37 formed longer myelin internodes than wild-type cells (Fig. 2i,j). In summary, these results suggest that *GPR37* negatively regulates the late-stage differentiation of oligodendrocytes.

### *Gpr37* ^*−/−*^ mice exhibit precocious- and hyper-myelination

**Figure 3:**
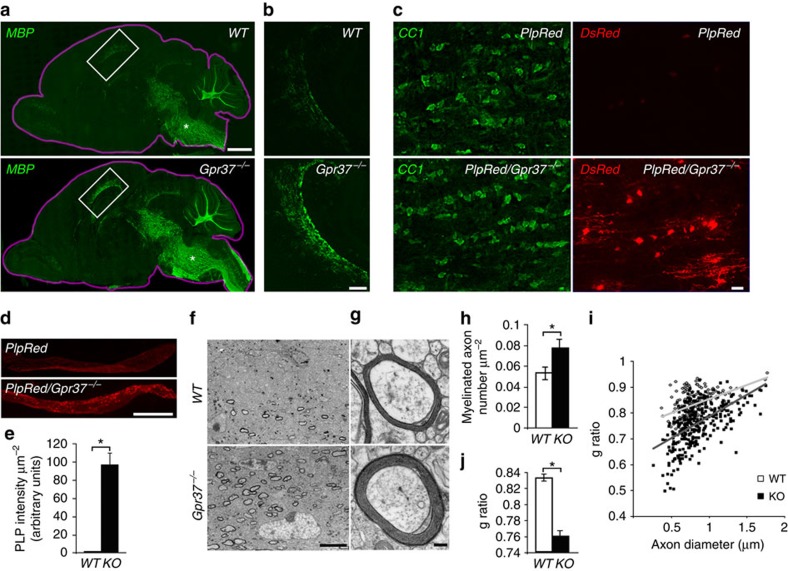
Absence of *Gpr37* results in precocious myelination *in vivo*. (**a**) Immunolabelling of *WT* and *Gpr37*^*−/−*^ P9 mice brains (delineated with a purple line) with an antibody to MBP. (**b**) Higher magnification of the corpus callosum (white rectangle in a). (**c**) Fluorescent images of longitudinal sections of optic nerves isolated from P12 *PlpRed* (upper panel) and *PlpRed/Gpr37*^*−/−*^ (lower panel) mice, immunolabelled with an antibody to APC (CC1). The CC1 (left) and red fluorescent (DsRed; right) signals are shown in separate panels. (**d**) Lower magnification images showing the distribution of DsRed in *PlpRed* (upper panel) and *PlpRed/Gpr37*^*−/−*^ (lower panel) optic nerves. (**e**) Fluorescence intensity of DsRed in optic nerves of the two genotypes (d) is shown per μm^2^ (*P<0.01, *n*=12 sections from 4 mice per each genotype). (**f**,**g**) Electron microscope images showing cross-sections through the corpus callosum of P14 *WT* (upper panels) and *Gpr37*^*−/−*^ (lower panels) mice. (**h**) The number of myelinated axons per μm^2^ was significantly higher in *Gpr37*^*−/−*^ than in *WT* (*P<0.05, 12 images from 3 WT and 8 images from 3 KO mice). (**i**,**j**) *Gpr37*^*−/−*^ exhibit significantly thicker myelin during development. G-ratio of myelinated axons in P14 corpus callosum is presented as a function of axon diameter (**i**), or as an average value (**j**). *Gpr37*^*−/−*^ exhibit a significantly lower g-ratio than *WT* mice (*WT*=155 axons, *Gpr37*^*−/−*^=219 axons from three mice of each genotype, *P<0.001, Student's *t*-test). Bars represent mean±s.e.m. Scale bars, (**a**) 1 mm; (**b**) 200 μm; (**c**) 10 μm; (**d**) 500 μm; (**f**) 5 μm; (**g**) 0.2 μm.

To examine whether the faster differentiation displayed by *Gpr37*^*−/−*^ oligodendrocytes affects myelination *in vivo*, we labelled sagittal sections of P9 mouse brains with an antibody to myelin basic protein (MBP) (Fig. 3a). Sagittal sections were used since myelination proceeds in a caudal (posterior) to rostral (anterior) direction during brain development. In *Gpr37*^*−/−*^, we detected intense myelin formation in rostral brain regions such as the corpus callosum, which only began to myelinate in wild-type mice at this developmental stage (Fig. 3a,b). Crossing the *Gpr37*^*−/−*^ mutant with a *PLP-dsRed* transgenic mouse (expressing a red fluorescence protein under the PLP promoter^37^) allowed us to compare the fluorescence signal between *PLP-dsRed* and *PLP-dsRed/Gpr37*^*−/−*^. In the optic nerve at P12, PLP-derived fluorescence signal was detected in CC1-positive oligodendrocytes in *PLP-dsRed/Gpr37*^*−/−*^ but not in *PLP-dsRed* control mice (Fig. 3c–e). Consistent with the role of GPR37 in late oligodendrocytes differentiation, neither the number of PDGFRα positive OPCs, nor their proliferation increased in *Gpr37*^*−/−*^ optic nerve (Supplementary Fig. 3). Electron microscopy analysis of corpus callosum at P14 revealed that *GPR37*^*−/−*^ mice exhibit a significantly higher number of myelinated axons (Fig. 3f,h), and thicker myelin profiles (that is, lower g-ratio) than their wild-type controls (Fig. 3g,i,j). These results show that the absence of GPR37 results in accelerated myelination.

**Figure 4:**
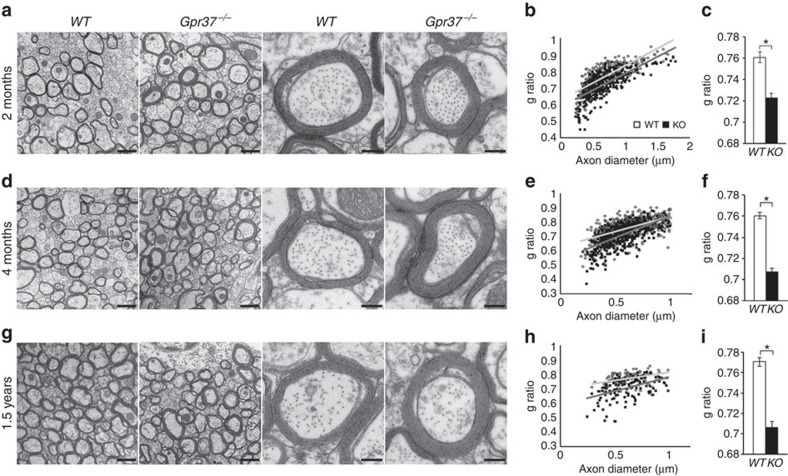
Absence of Gpr37 results in hypermyelination of the corpus callosum. (**a**,**d**,**g**) Electron micrographs of midsagittal sections of the corpus callosum from *WT* and *Gpr37*^*−/−*^ mice at the age of 2 months (**a**–**c**), 4 months (**d**–**f**) and 1.5 years (**g**–**i**). Representative higher magnification images are shown on the right columns. (**b**,**e**,**h**) g-ratio as a function of axonal diameter. *GPR37*^*−/−*^ shows lower g-ratio than *WT*. Total of 274 axons (WT) and 373 axons (KO) from three mice (for 2 month), 481 axons (WT) and 497 axons (KO) from 3 mice (for 4 month), 97 axons (WT)and 139 axons (KO) from 2 mice (for 1.5 year) of each genotype were analysed. (**c**,**f**,**i**) The averaged g-ratio of *Gpr37*^*−/−*^ is significantly lower than *WT* (*P<0.001). Scale bars, 1 μm (left two panels) and 0.2 μm (right two panels). Bars represent mean±s.e.m.

To determine whether the advanced myelination detected during development in *Gpr37*^*−/−*^ mice persisted into adulthood, we analysed the corpus callosum of wild-type and mutant mice at 2, 4 and 18 months of age by electron microscopy (Fig. 4). At all ages examined, we noted an increase in myelin thickness with no increase in the diameter of myelinated axons. This was reflected by a significant decrease in g-ratio in *Gpr37*^*−/−*^ compared with wild-type mice (Fig. 4b,c,e,f,h,i). Average g-ratios obtained were *WT* 0.76±0.005 versus *Gpr37*^*−/−*^ 0.72±0.004 at 2 months; *WT* 0.76±0.003 versus *Gpr37*^*−/−*^ 0.70±0.003 at 4 months; and *WT* 0.77±0.004 versus *Gpr37*^*−/−*^0.70±0.006 at 1.5 years of age. The reduction in g-ratio in *Gpr37*^*−/−*^ brains was also observed in spinal cords of mice of the same ages (Supplementary Fig. 4). These results demonstrate that the absence of GPR37 results in hypermyelination, further supporting its role as a negative regulator of myelination in the CNS.

### GPR37 signalling is mediated by ERK

**Figure 5:**
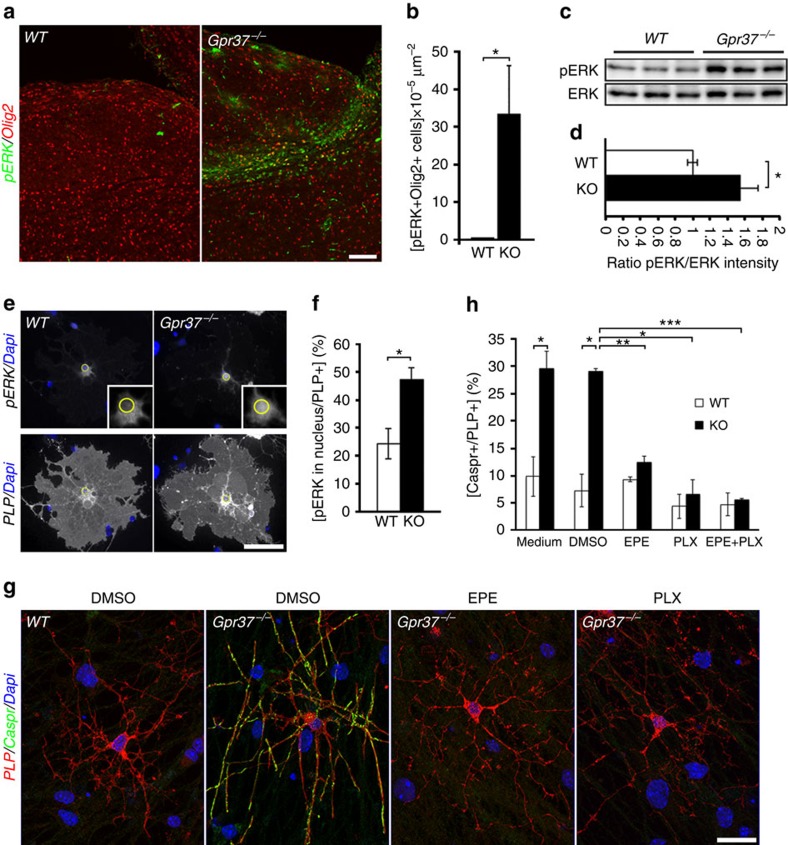
Gpr37-dependent inhibition of oligodendrocyte differentiation is mediated by ERK phosphorylation and nuclear translocation. (**a**) Immunolabelling of sagittal brain sections of P12 brain stems from wild type (WT) and KO (*Gpr37*^*−/−*^) mice using antibodies to phosphorylated ERK (pERK) and Olig2. (**b**) The number of pERK and Olig2 positive cells in *Gpr37*^*−/−*^ compared with WT brainstem (*P<0.05, *n*=5 images from two mice per genotype). (**c**) Western blot analysis of purified OPC cultures at DIV7 (three cultures per each genotype are shown). Blots were incubated with antibodies to pERK or general ERK (ERK) as indicated. (**d**) Relative value of pERK intensity normalized by general ERK (*p*=0.02, *n*=7 cultures each genotype). (**e**) pERK is present in the nucleus of oligodendrocytes lacking Gpr37) OPC cultures isolated from wild-type (WT) or KO (*Gpr37*^*−/−*^) mice were fixed at DIV5 and immunolabelled using antibodies to pERK, PLP and Dapi. PLP and ERK immunoreactivity are shown in separate panels along with the Dapi signal. The location of the nucleus is marked with a yellow circle in the high magnification of the boxed area (insets). (**f**) Percentage of PLP-positive oligodendrocytes showing nuclear localization of pERK in wild-type (WT) and KO cultures. (*P<0.05, *n*=3 different primary cultures for each genotype; 100 PLP-positive oligodendrocytes were counted per each culture). (**g**,**h**) ERK signaling mediates Gpr37 effects on oligodendrocyte differentiation. (**g**) OPCs isolated from *Gpr37*^*−/−*^ or WT mice as indicated were grown with wild-type DRG neurons. Co-cultures were grown for 1 day in their growth medium and maintained for 6 days in a medium containing 10 μM EPE, 1 μM PLX4032 or DMSO as control before fixing and labelling with antibodies to Caspr and PLP. Nuclei were labelled with Dapi. (**h**) Quantitation of the results showing the fold change in the per cent of PLP-positive cells that associate with axonal Caspr immunoreactivity in all samples compare to the non-treated wild-type cultures. *Gpr37*^*−/−*^ oligodendrocytes treated with ERK inhibitors showed a significantly low number of Caspr-positive axons, when compared with DMSO-control (*P<0.05, **P<0.01, ***P<0.001; *n*=3 different primary cultures for each genotype, 100 PLP-positive cells were counted in each treatment). Bars represent mean±s.e.m. Scale bars, (**a**) 100 μm; (**e**) 50 μm; and (**h**) 20 μm.

Previous studies have shown that the extracellular signal-regulated kinase (ERK1/2)/MAPK pathway regulates oligodendrocyte development and myelin thickness^38^. Given the role this signalling system plays in mediating the action of GPCRs^39^, we sought to compare ERK1/2 phosphorylation in wild-type and *Gpr37*^*−/−*^ brains. Immunofluorescence labelling of P12 brainstem sections using antibodies to Olig2 and phosphorylated ERK1/2, revealed an intense phospho-ERK immunoreactivity in the mutant, but not in the wild-type controls (Fig. 5a,b). A significant increase in ERK phosphorylation was also evident by western blot analysis of cultured oligodendrocytes lacking GPR37 (Fig. 5c,d). As one of the key steps in ERK1/2 signalling is their nuclear translocation^40^, we immunolabelled cultured oligodendrocytes isolated from wild-type or *Gpr37*^*−/−*^ mice using antibodies to PLP and phospho-ERK1/2 (Fig. 5e). We noted that the number of *Gpr37*^*−/−*^ oligodendrocytes exhibiting nuclear localization of pERK1/2 was double than the wild-type cells (Fig. 5f). These results indicate that the absence of GPR37 results in enhanced ERK1/2 phosphorylation and translocation to the nucleus.

To assess whether the effect of GPR37 on oligodendrocyte differentiation is mediated by MAPK signalling, we treated myelinating co-cultures containing wild-type or *Gpr37*^*−/−*^ oligodendrocytes with PLX4032 (ref. 41), an inhibitor of Raf kinases, which relay extracellular signals to the MAPK module and play a role in oligodendrocyte maturation and myelination^42^. As an alternative approach, the cultures were grown in the presence of myr-EPE, a myristoylated phosphomimetic peptide that inhibits the nuclear translocation of ERK1/2 (ref. 40). This peptide also prevented the accumulation of ERK1/2 in the nucleus of oligodendrocytes lacking GPR37 (Supplementary Fig. 5a). Cells were immunolabelled for PLP and Caspr (Fig. 5h). The clustering of axonal Caspr induced by oligodendrocyte contact was used to evaluate differentiation. Remarkably, both inhibitors completely abolished the number of PLP-positive oligodendrocytes that were associated with intense axonal clustering of Caspr in *Gpr37*^*−/−*^ (Fig. 5g). PLX4032 and myr-EPE had no effect on wild-type cultures (Fig. 5g), nor did they affect the number of Olig2 cells of both genotypes (Supplementary Fig. 5b). In line with previous studies suggesting that GPR37 and GPR37L1 are coupled to Gα_i/o_ (that is, inhibiting adenylate cyclase)^43^, we detected an increase in cAMP levels in both *Gpr37*^*−/−*^ brains (Supplementary Fig. 6a) and cultured *Gpr37*^*−/−*^ oligodendrocytes (Supplementary Fig. 6b). Furthermore, we noted a significant decrease in the number of *Gpr37*^*−/−*^ oligodendrocytes displaying nuclear localization of ERK1/2 after treatment with either an adenylate cyclase (SQ 22536) (Supplementary Fig. 6c,d) or an EPAC (exchange protein activated by cAMP) (ESI-09) inhibitor (Supplementary Fig. 7), indicating that cAMP signalling downstream of GPR37 is mediated by EPAC. As an additional indication for the involvement of cAMP in GPR37 signalling, we found that the addition of the phosphodiesterase inhibitor 3-isobutyl-1-methylxanthine (IBMX) enhances the differentiation of wild-type oligodendrocyte to a level comparable to that observed in *Gpr37*^*−/−*^ cells (Supplementary Fig. 8).

As the differentiation of the oligodendrocytes is controlled by a complex transcriptional network^44^,^45^, we examine whether the absence of GPR37 affects the expression of 12 transcription factors. We performed qRT-PCR of P4 brainstem RNA isolated from *Gpr37*^*−/−*^ and their littermate wild-type control mice (Supplementary Fig. 9). This analysis revealed that the absence of GPR37 lead to increased expression of the stimulatory myelin regulatory factor (Myrf), which activates myelin gene expression genes^46^,^47^,^48^. It also resulted in a decrease in the level of the inhibitory bHLH factor Hes5 (ref. 49), which negatively regulates Myrf^50^. Taken together, our results indicate that the inhibitory action of GPR37 in oligodendrocytes is mediated by the suppression of cAMP-dependent inhibition of ERK1/2 phosphorylation, its nuclear translocation and attenuation of Myrf expression.

## Discussion

The generation of myelinating oligodendrocytes from proliferating OPCs during development involves distinct differentiation steps defined by cellular morphology and the combinatorial expression of specific marker proteins^34^. After exiting the cell cycle, OPCs first differentiate into pre-myelinating oligodendrocytes, and later on to mature myelinating oligodendrocytes. Although several signalling systems and receptors were shown to control OPC proliferation and their early differentiation into pre-myelinating oligodendrocytes^8^, it is unclear whether additional signals are required for the progression of the latter into myelin forming cells^1^. In the present study, we identify GPR37 as a negative regulator of oligodendrocyte maturation (that is, late-stage differentiation) and myelination. Its role in myelination is specific to the CNS as it is not present in myelinating Schwann cells, further supporting the notion that CNS and PNS myelination are regulated by different signalling systems^51^. The following findings indicate a role for GPR37 in the development of the oligodendrocyte lineage: (i) GPR37 is highly enriched in oligodendrocytes compared with any other cell types in the CNS. (ii) Its expression begins relatively late during development after pre-myelinating oligodendrocytes have already formed. (iii) OPC proliferation or early differentiation into O4-positive cells is not regulated by GPR37. (iv) Absence of GPR37 enhances the differentiation of pre-myelinating oligodendrocytes into myelin producing cells. (v) Genetic deletion of *Gpr37* in mice results in precocious differentiation of oligodendrocytes during development and a premature appearance of myelin in rostral areas of the brain. (vi) *Gpr37*^*−/−*^ exhibits hypermyelination during development that last in the adult CNS. (vii) The inhibitory effect of GPR37 is mediated by suppression of ERK1/2, a signalling pathway known to control myelin thickness in the CNS (refs 38, 52). Although we have not used a cell-type-specific knockout mouse in our studies, the observation that *Gpr37*^*−/−*^ oligodendrocytes cultured with wild-type neurons exhibit precocious differentiation reveals that the function of GPR37 in oligodendrocytes is cell autonomous. This conclusion is also supported by the observation that shRNA knockdown of Gpr37 in oligodendrocytes results in their enhanced differentiation.

**Figure 6:**
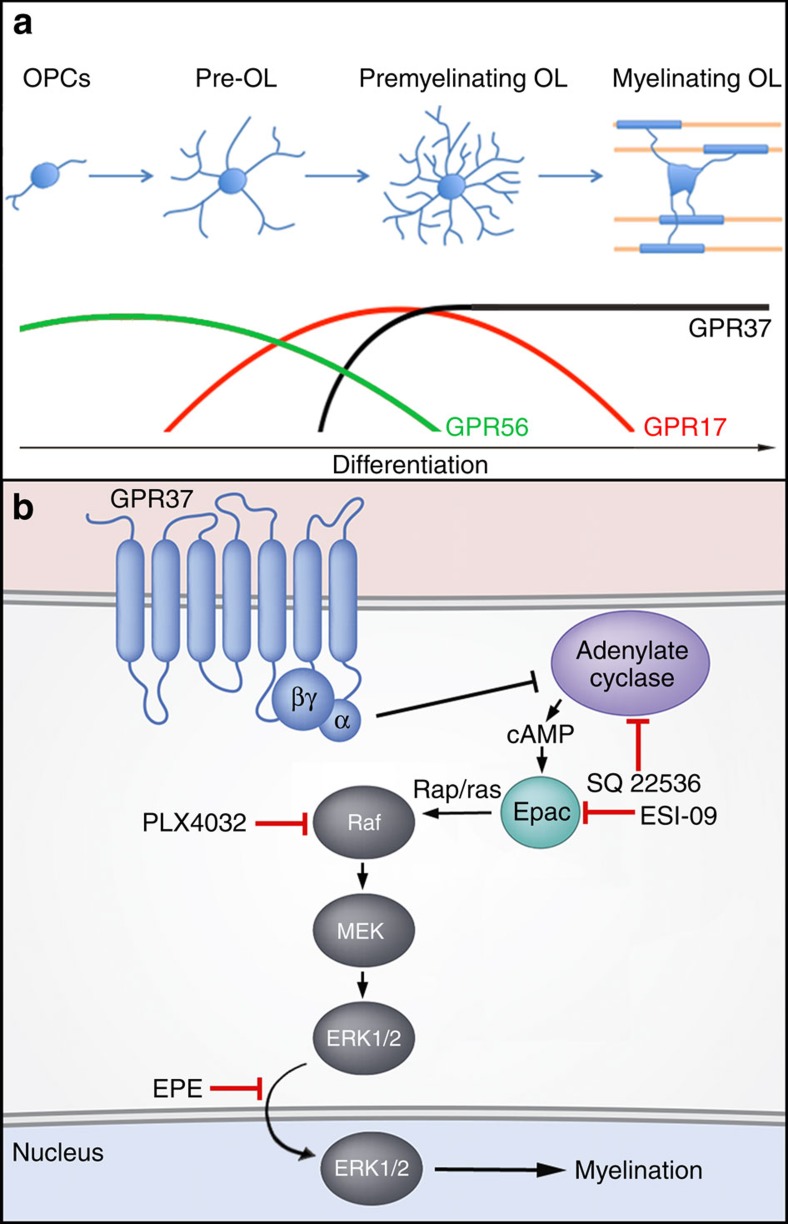
GPR37 regulates oligodendrocyte myelination through MAPK signaling. (**a**) Sequential expression and activity of GPCRs during differentiation of the oligodendrocyte lineage. GPR56 regulates OPC proliferation, while GPR17 and GPR37 negatively regulate two consecutive stages of oligodendrocytes differentiation. (**b**) A schematic model depicting GPR37 signalling. Relief of GPR37 inhibition results in increase in cAMP and Epac-dependent activation of MAPK cascade, resulting in translocation of phospho-ERK1/2 into the nucleus and myelination. Red lines mark the point of action of the various pharmacological inhibitors used.

Previous studies have shown that GPR17 negatively regulates oligodendrocyte differentiation and myelination^12^,^53^. GPR17 is expressed at the transition from late OPCs (also termed pre-oligodendrocytes) to immature pre-oligodendrocytes, but is downregulated in myelinating oligodendrocytes^12^,^54^. Such a pattern of expression is distinct from GPR37, which appears in pre-myelinating oligodendrocytes, and continues to be expressed in myelinating oligodendrocytes in the adult. Moreover, double-labelling experiments of brainstem at P12, revealed that the number of cells expressing both GPR17 and GPR37 is rather limited and account for only 5% of pre-myelinating oligodendrocytes. This difference is also reflected by the different phenotypes obtained by deleting these GPCRs in mice; while the absence of either GPR17 or GPR37 resulted in premature formation of myelinating oligodendrocytes during development, myelination of adult *GPR17*^*−/−*^ mice was comparable to wild type, whereas the absence of GPR37 results in persistent hypermyelination. These results suggest that although both GPR17 and GPR37 negatively regulate oligodendrocyte differentiation, they act at different stages of development of the lineage. Interestingly, in addition to GPR17 and GPR37, it was recently shown that the adhesion-type GPR56 affects oligodendrocyte differentiation by positively regulating OPCs proliferation^21^,^22^. Overexpression of GPR56 causes an increase in the number of OPCs and thus inhibition of myelination^21^, whereas its absence causes a reduction in the number of OPCs, and as a result premature differentiation and hypomyelination^21^,^22^. Given the differential expression of GPR56, GPR17 and GPR37 during development, as well as the unique role they play in regulating OPCs proliferation (GPR56), early (GPR17) and late (GPR37) stages of oligodendrocyte differentiation, we propose that the development of the oligodendrocyte lineage from OPCs to myelinating cells is controlled by the sequential activity of inhibitory GPCRs (Fig. 6a). The regulation of CNS myelination by inhibitory signals^55^,^56^ may be necessary due to the intrinsic ability of oligodendrocytes to wrap permissive substrates such as fixed axons^57^ and inert nanofibres^58^. Our results also suggest that although the terminal differentiation of OPCs and subsequent myelination are tightly coupled and are thought to proceed rapidly and by default^59^,^60^, they are controlled by two temporally distinct inhibitory signals. Such a multi-checkpoint mechanism may ensure that the correct axons are myelinated during development and would allow myelin plasticity in the adult. As recently noted^8^, this would be of further importance in humans, where pre-myelinating oligodendrocytes may persist for a long period of time before myelinating^61^. An open question for future studies would be to determine the extracellular signals regulating the activity of GPR37 in myelination. In this regard, it should be noted that prosaposin, which was previously identified as a ligand for GPR37 (refs 43, 62), enhances OPC differentiation independently of the presence of GPR37 (Supplementary Fig. 10), suggesting that it is not the major ligand controlling the action of this receptor in oligodendrocytes. In line with this notion, oligodendrocytes express two additional prosaposin receptors, namely GPR37L1 and low-density lipoprotein receptor-related protein 1 (LRP1) (ref. 30). Gain- and loss-of-function studies have linked ERK1/2 signalling to the regulation of myelin thickness in both CNS^38^,^52^ and PNS^63^. Entirely consistent with these results, we found that the absence of GPR37 caused an increase in ERK1/2 phosphorylation in both cultured oligodendrocytes and mouse brains. Pharmacological inhibition of Raf kinases, which are required for the sequential activation of MEK1/2 and ERK1/2 (ref. 39), prevented the premature differentiation of *Gpr37*^*−/−*^ oligodendrocytes without affecting their proliferation. Furthermore, a similar effect was obtained by treatment of *Gpr37*^*−/−*^ oligodendrocytes with the myr-EPE peptide, which inhibits the nuclear translocation of ERK1/2 (ref. 40). Nuclear translocation of ERK1/2 in *Gpr37*^*−/−*^ oligodendrocytes was also attenuated by pharmacological inhibition of adenylate cyclase, suggesting that GPR37 signalling is normally suppresses cAMP levels. This notion is supported by the observation that oligodendrocytes and mouse brains lacking GPR37 contain higher levels of cAMP compared with their wild-type controls. In line with these observations, cAMP was shown to stimulate the expression of myelin genes such as MBP and PLP, and the maturation of oligodendrocytes^64^. Similarly, inhibition of the cAMP-hydrolyzing enzyme phosphodiesterase 4 (Pde4) promoted MAPK signalling and OPC differentiation^65^,^66^. Overall, these results suggest that GPR37 controls late-stage oligodendrocyte differentiation, at least in part, by suppressing cAMP, which further leads to the downstream inhibition of ERK1/2 activation and nuclear translocation (Fig. 6b).

Development of the oligodendrocytes lineage is controlled by an intricate regulatory transcriptional network that includes multiple inhibitory and stimulatory factors^44^,^45^. We found that the absence of Gpr37 lead to increase expression of myelin regulatory factor (Myrf), a key transcription factor that present in promyelinating oligodendrocytes (but not OPCs) and is required for myelination, as well as for myelin maintenance in the adult by controlling the expression of myelin genes^46^,^47^,^48^. This result suggests that by suppressing ERK activation and nuclear translocation, Gpr37 negatively regulates the expression of Myrf. In line with this idea, it was recently found that Myrf transcript decreases in mice lacking ERK1/2 in oligodendrocytes, and conversely, increases in mice expressing a constitutively active Mek1 transgene^67^. In addition, the absence of GPR37 resulted in a significant decrease in the level of the inhibitory bHLH protein Hes5 that negatively regulate myelin gene transcription^49^. Notably, Hes5 also negatively regulates the transcription of the promyelinating factor SOX10 that activates Myrf (ref. 50).

In summary, we identify GPR37 as a negative regulator of CNS myelination by controlling the transition from pre-myelinating oligodendrocytes to myelin forming cells. As such, our findings may have potential therapeutic implications for demyelinating and other neurological diseases involving white matter pathologies.

## Methods

### Mice

*Gpr37*^*+/−*^ (B6.129P2*-Gpr37*^*tm1Dgen*^/J) mice were obtained from the Jackson Laboratory (http://jaxmice.jax.org/strain/005806.html) and were maintained on a C57BL/6J background. Age of mice used for experiments is detailed in the figure legends; male and female littermate mice were used interchangeably. Mice were genotyped for the targeted allele by PCR using tail DNA. For the targeted allele, the forward primer in neo 5′-gggtgggattagataaatgcctgctct-3′ and the gene-specific reverse primer 5′-ggccaagagagaattggagatgctc-3′ were used. For the endogenous allele, the gene-specific forward primer 5′-aacgggtctgcagatgactgggttc-3′ and the gene-specific reverse primer 5′-ggccaagagagaattggagatgctc-3′ were used. Transgenic PLP-dsRed mice^37^ were kindly obtained from Drs Frank Kirchhoff and Klaus Armin Nave. All experiments were performed in compliance with the relevant laws and institutional guidelines, and were approved by the Weizmann Institute's Animal Care and Use Committee.

### RT–PCR and qRT-PCR

Total RNA was isolated with TRIzol reagent (Sigma) from freshly dissected mouse brains or cultured cells. Isolated RNA was treated with DNaseI to eliminate genomic DNA before reverse transcription. Mouse cDNAs were prepared using SuperScript II Reverse Transcriptase (Invitrogen). Specific primer sets were used for GPR37 (forward, 5′-ACACAGGTGTGATTGAAGAAGC-3′; reverse, 5′-ATAGTACTGAAGGGCGACAGC-3′), GPR37L1 (forward, 5′-CTTTAGGTGGGCATAGAGC-3′; reverse, 5′-TGGAGAACTGGTTGATGAGGC-3′), MAG (forward, 5′-TGCCGCTGTTTTGGATAA-3′; reverse, 5′-CGCCTCGGAAATAGTATTTG-3′), MBP (forward, 5′-CCAGAGCGGCTGTCTCTTCC-3′; reverse, 5′-CATCCTTGACTCCATCGGGCGC-3′) and actin (forward, 5′-GAGCACCCTGTGCTGCTCACCGAGG-3′; reverse, 5′-GTGGTGGTGAAGCTGTAGCCACGCT-3′). qRT-PCR was performed in the PCR 7900HT Real-Time PCR System (Applied Biosystems) using SYBR Green mix. The following primers were used: GPR37 (forward, 5′-CCTGCAAGATCGTGCCCTA-3′; reverse, 5′-AGTACATCTGGACGTTGGTGG-3′), GPR17 (forward 5′-CAGCTACGAGGAGTCCACCTGGAGCAC-3′; reverse, 5′-CGGTAGGGCTGCCTCCAGACCGTTCAT-3′), Hes5 (forward, 5′-AACTCCAAGCTGGAGAAGGC-3′; reverse, 5′-GTCAGGAACTGTACCGCCTC-3′), Hes1 (forward, 5′-AATGACTGTGAAGCACCTCC-3′; reverse, 5′-ATTTCCCCAACACGCTCGG-3′), Id2 (forward, 5′-CCTGCATCACCAGAGACCTG-3′; reverse, 5′-TTCGACATAAGCTCAGAAGGGAA-3′), Tcf4 (forward, 5′-CATCACCAACAGCGAATGGC-3′; reverse, 5′-CACTGCTTACAGGAGGCGAA-3′), Ascl1 (forward, 5′-CAACCGGGTCAAGTTGGTCA-3′; reverse, 5′-CTCATCTTCTTGTTGGCCGC-3′), Olig1 (forward, 5′-GCTCGCCCAGGTGTTTTGT-3′; reverse, 5′-GCATGGAACGTGGTTGGAAT-3′), Olig2 (forward, 5′-AGAGCCAGGTTCTCCTCCG-3′; reverse, 5′-ACTAGACACCAGGCTGGCGT-3′), Nkx2.2 (forward, 5′-GGTTCCAGAACCATCGCTACA-3′; reverse, 5′GCTTCGATCCTGGCATCCAT3′), Nkx6.2 (forward, 5′-CATGACCGAGAGCCAAGTGA-3′; reverse, 5′-GCTTCTTTTTAGCCGACGCC-3′), Sox10 (forward, 5′-AGCCCAGGTGAAGACAGAGA-3′; reverse, 5′-AGTCAAACTGGGGTCGTGAG-3′), Yy1 (forward, 5′-TTGAGCTCTCAACGAACGCTTTGC-3′; reverse, 5′-TCAGACCCTAAGCAACTGGCAGAA-3′), and Myrf (forward, 5′-TGGCAACTTCACCTACCACA-3′; reverse, 5′-GTGGAACCTCTGCAAAAAGC-3′). GAPDH (forward, 5′-GGTCGGTGTGAACGGATTTG-3′; reverse, 5′-TCGTTGATGGCAACAATCTCCACT-3′) was used as reference genes. All reactions were carried out in triplicate and GAPDH was used for normalization.

### shRNA

Oligonucleotides used for the generation of the pSUPER retroviral vectors were as follows: GPR37-sh1 sense, 5′-gatctccccaacgtccagatgtactattcaagagatagtacatctggacgttggtttttc-3′; GPR37-sh1 antisense, 5′-tcgagaaaaaccaacgtccagatgtactatctcttgaatagtacatctggacgttgggga-3′; GPR37-sh2 sense, 5′-gatctccgaaggccagtacccgtggattcaagagatccacgggtactggccttctttttc-3′; and GPR37-sh2 antisense, 5′-tcgagaaaaagaaggccagtacccgtggatctcttgaatccacgggtactggccttcgga-3′. Retroviruses were produced by transfection of Phoenix cells.

### *In situ* hybridization

Synthetic digoxigenin-labelled riboprobes (cRNA) were produced based on a pcDNA3 plasmid containing corresponding base pairs of the indicated mRNA. *In vitro* transcription was done from both sides with either SP6 or T7 RNA polymerase, generating antisense or sense (control) cRNA probes. The probes were alkaline hydrolyzed to an average length of 200–400 bases. *In situ* hybridization was performed using cRNA probes for GPR37 on adult rat brain cryosections as previously described^27^.

### LacZ staining

For LacZ staining, mice were anaesthetised with ketamine/xylazine, injected intraperitoneally and perfused with 1% paraformaldehyde (PFA) (pH 7.4). Tissues were collected and post-fixed with 1% PFA in 30% sucrose/ PBS overnight at 4 °C, followed by two hours of fixation with 0.5% glutaraldehyde in 30% sucrose/ PBS at 4 °C. Tissues were washed with 30% sucrose/ PBS. Brains were embedded in OCT and sectioned (40-μm sections) on a freezing microtome, and sections were stored free-floating in PBS. Samples were washed once with wash solution (20 mM Tris-HCl (pH 7.3), 0.01% sodium deoxycholate, 0.02% NP40 and 2 mM MgCl_2_) and immediately used for LacZ staining. LacZ staining was performed overnight at 37 °C in staining solution (20 mM Tris-HCl (pH 7.3), 5 mM potassium ferrocyanide, 5 mM potassium ferricyanide, 0.01% sodium deoxycholate, 0.02% NP40, 2 mM MgCl_2_ and 1 mg ml^−1^ X-gal). Tissues were washed once in wash solution, transferred to glass slides, and mounted with mounting medium. Optic nerves, sciatic nerves and spinal cords were stained as a whole mount after fixation.

### Antibodies and reagents

The following antibodies were used: mouse monoclonal antibodies to β gal (1:1,000, G8021, Sigma), pERK (1:50, sc-7383, Santa Cruz), CC1 (1:50, OP80, Millipore), neurofilament (1:1,000, NE1017, Millipore), and rabbit polyclonal antibodies to Olig2 (1:500, AB9610, Millipore), ki67 (1:500, SP6, Cell Marque), Caspr (1:1,000) (ref. 68), pERK (1:1,000, 4370P, Cell Signaling), ERK (1:2,000, M5670, Sigma), GPR17 (1:20, Cayman Chemical, 17087), and goat polyclonal antibody to GPR37 (1:50, Santa Cruz, sc-27548), and rat monoclonal antibodies to MBP (1:300, MAB386, Chemicon), PDGFRα (1:1,000, APA5, BD Pharmingen), and hybridoma supernatants of mouse anti-O4 (1:5) and rat anti-PLP (1:10, AA3). Secondary antibodies were obtained from Jackson Immunoresearch and Invitrogen. PLX4032 (ref. 41) and myr-EPE (ref. 40) are generous gifts from Prof. Rony Seger (Weizmann Institute of Science, Rehovot, Israel). SQ 22536, IBMX and ESI-09 were purchased from Sigma. cAMP levels were determined by ELISA according to the manufacturer's protocol (cAMP ELISA kit, Enzo Life Sciences).

### Immunofluorescence and immunoblotting

For immunohistochemistry, mice were anaesthetized and perfused with 2% PFA/PBS. Brains and optic nerves were isolated and post-fixed on ice for 15 min, followed by 30% sucrose/PBS at 4 °C overnight. Tissues were embedded in OCT and sectioned. Sections were permeabilized in methanol at −20 °C for 5 min. Immunostaining of GPR37 was carried out using 2%PFA-perfused mouse brain sections that were permeabilized with cold acetone and blocked with 5% fish gelatin and 0.5% Triton-X100 at RT for 1 h. For immunocytochemistry, cells were fixed with 4% PFA/PBS for 15 min at room temperature. For O4 staining, live cells were incubated with medium containing the O4 antibody at 37 °C for 45 min before fixation. For both immunohistochemistry and immunocytochemistry, samples were washed with PBS and blocked with PBS containing 5% normal goat serum, 0.5% Triton X-100, 0.05% sodium azide for 1 h. Samples were incubated overnight at 4 °C with primary antibodies diluted in blocking solution, washed three times in PBS, incubated for 1 h with secondary antibodies, washed in PBS and mounted with Elvanol. Images were obtained using an Axio Imager Z1 equipped with Apotome (Carl Zeiss), LSM700 confocal microscope (Carl Zeiss), or Pannoramic digital slide scanner (3DHISTECH). For image analysis, images were taken in equivalent spatial distribution from all slides. Image analysis was performed using Volocity 4.2.1 image analysis software (Perkin-Elmer) and ZEN 2011 software (Carl Zeiss). Western blot analysis was done using OPC cultures lysed in loading buffer and chemiluminescence was detected using the ChemiDoc MP System (Bio-Rad). Image (Fig. 5c) has been cropped for presentation (full-size image is shown in Supplementary Fig. 10.

### Cell cultures

For co-cultures, mouse DRG neuronal cultures and mouse glia mixed cultures of each genotype were prepared separately in advance. DRG neurons were prepared as described previously^69^. Glial mixed cultures were prepared from P0-P2 mouse cortices on PDL-coated flasks and maintained in DMEM/F-12 containing 10% fetal bovine serum, 5% horse serum and penicillin-streptomycin. After 9–10 days, oligodendrocytes isolated by shaking were seeded on DRG neuronal cultures and maintained in coculture medium (DMEM containing B-27 supplement, N-2 supplement, 5 μg ml^−1^ N-Acetyl-Cysteine, 5 μM forskolin and penicillin-streptomycin). The medium was changed every other day. OPC cultures were prepared as described above on PLD and poly-L-ornithine-coated coverslips. OPC cultures were maintained in Sato medium (DMEM containing B-27 supplement, Glutamax, penicillin-streptomycin, 1% horse serum, sodium pyruvate, 0.34 μg ml^−1^ T3 and 0.4 μg ml^−1^ T4). For the first 2 days, PDGF (final 25 ng ml^−1^) was added to the Sato medium.

### Electron microscopy

Mice were anaesthetised and perfused with a fixative containing 4% PFA, 2.5% glutaraldehyde and 0.1 M cacodylate. Brains and spinal cords were isolated and incubated in the fixative overnight at room temperature and processed as previously described^68^. Samples were examined using a Philips CM-12 transmission electron microscope. The EM micrographs were analysed using computer-assisted analysis software (analysis, Soft Imaging System) for axon diameter and total outer axon diameter containing myelin. G-ratio was calculated by dividing the measured inner axonal diameter to the measured total outer axonal diameter.

### Statistical analyses

All graph data are presented as the mean±s.e.m. Statistical analyses were performed using an unpaired Student's *t*-test with two tails, unequal variance. Images were scored blinded to genotype before quantification. For *in vivo* experiments and *in vitro* cultures, mice were prepared as pairs before the experiments. Sample size was not predetermined, but was based on similar studies in the field.

## Additional information

**How to cite this article:** Yang, H.-J. *et al.* G protein coupled receptor 37 is a negative regulator of oligodendrocyte differentiation and myelination. *Nat. Commun.* 7:10884 doi: 10.1038/ncomms10884 (2016).

## Supplementary Material

Supplementary Information Supplementary Figures 1-10

## References

[b1] Nave K. A. & Werner H. B. Myelination of the nervous system: mechanisms and functions. Annu. Rev. Cell Dev. Biol. 30, 503–533 (2014). 25288117 10.1146/annurev-cellbio-100913-013101

[b2] Liu J. *et al.* Impaired adult myelination in the prefrontal cortex of socially isolated mice. Nat. Neurosci. 15, 1621–1623 (2012). 23143512 10.1038/nn.3263 PMC3729624

[b3] Young K. M. *et al.* Oligodendrocyte dynamics in the healthy adult CNS: evidence for myelin remodeling. Neuron 77, 873–885 (2013). 23473318 10.1016/j.neuron.2013.01.006 PMC3842597

[b4] Perlman S. J. & Mar S. Leukodystrophies. Adv. Exp. Med. Biol. 724, 154–171 (2012). 22411242 10.1007/978-1-4614-0653-2_13

[b5] Nave K. A. & Ehrenreich H. Myelination and oligodendrocyte functions in psychiatric diseases. JAMA Psychiatry 71, 582–584 (2014). 24671770 10.1001/jamapsychiatry.2014.189

[b6] Franklin R. J. & Gallo V. The translational biology of remyelination: past, present, and future. Glia 62, 1905–1915 (2014). 24446279 10.1002/glia.22622

[b7] Hughes E. G., Kang S. H., Fukaya M. & Bergles D. E. Oligodendrocyte progenitors balance growth with self-repulsion to achieve homeostasis in the adult brain. Nat. Neurosci. 16, 668–676 (2013). 23624515 10.1038/nn.3390 PMC3807738

[b8] Mitew S. *et al.* Mechanisms regulating the development of oligodendrocytes and central nervous system myelin. Neuroscience 276, 29–47 (2014). 24275321 10.1016/j.neuroscience.2013.11.029

[b9] Charles P. *et al.* Negative regulation of central nervous system myelination by polysialylated-neural cell adhesion molecule. Proc. Natl Acad. Sci. USA 97, 7585–7590 (2000). 10840047 10.1073/pnas.100076197 PMC16589

[b10] Fancy S. P. *et al.* Dysregulation of the Wnt pathway inhibits timely myelination and remyelination in the mammalian CNS. Genes Dev. 23, 1571–1585 (2009). 19515974 10.1101/gad.1806309 PMC2704469

[b11] Mi S. *et al.* LINGO-1 negatively regulates myelination by oligodendrocytes. Nat. Neurosci. 8, 745–751 (2005). 15895088 10.1038/nn1460

[b12] Chen Y. *et al.* The oligodendrocyte-specific G protein-coupled receptor GPR17 is a cell-intrinsic timer of myelination. Nat. Neurosci. 12, 1398–1406 (2009). 19838178 10.1038/nn.2410 PMC2783566

[b13] Wang S. *et al.* Notch receptor activation inhibits oligodendrocyte differentiation. Neuron 21, 63–75 (1998). 9697852 10.1016/s0896-6273(00)80515-2

[b14] Colognato H. *et al.* CNS integrins switch growth factor signalling to promote target-dependent survival. Nat. Cell Biol. 4, 833–841 (2002). 12379866 10.1038/ncb865

[b15] Xiao J. *et al.* Brain-derived neurotrophic factor promotes central nervous system myelination via a direct effect upon oligodendrocytes. Neurosignals 18, 186–202 (2010). 21242670 10.1159/000323170

[b16] Furusho M., Dupree J. L., Nave K. A. & Bansal R. Fibroblast growth factor receptor signaling in oligodendrocytes regulates myelin sheath thickness. J Neurosci. 32, 6631–6641 (2012). 22573685 10.1523/JNEUROSCI.6005-11.2012 PMC3367512

[b17] Ahrendsen J. T. & Macklin W. Signaling mechanisms regulating myelination in the central nervous system. Neurosci. Bull. 29, 199–215 (2013). 23558589 10.1007/s12264-013-1322-2 PMC4395498

[b18] Mogha A. *et al.* Gpr126 functions in Schwann cells to control differentiation and myelination via G-protein activation. J. Neurosci. 33, 17976–17985 (2013). 24227709 10.1523/JNEUROSCI.1809-13.2013 PMC3828454

[b19] Monk K. R. *et al.* A G protein-coupled receptor is essential for Schwann cells to initiate myelination. Science 325, 1402–1405 (2009). 19745155 10.1126/science.1173474 PMC2856697

[b20] Trimarco A. *et al.* Prostaglandin D2 synthase/GPR44: a signaling axis in PNS myelination. Nat. Neurosci. 17, 1682–1692 (2014). 25362470 10.1038/nn.3857

[b21] Ackerman S. D., Garcia C., Piao X., Gutmann D. H. & Monk K. R. The adhesion GPCR Gpr56 regulates oligodendrocyte development via interactions with Galpha12/13 and RhoA. Nat. Commun. 6, 6122 (2015). 25607772 10.1038/ncomms7122 PMC4302765

[b22] Giera S. *et al.* The adhesion G protein-coupled receptor GPR56 is a cell-autonomous regulator of oligodendrocyte development. Nat. Commun. 6, 6121 (2015). 25607655 10.1038/ncomms7121 PMC4302951

[b23] Piao X. *et al.* Genotype-phenotype analysis of human frontoparietal polymicrogyria syndromes. Ann. Neurol. 58, 680–687 (2005). 16240336 10.1002/ana.20616

[b24] Deshmukh V. A. *et al.* A regenerative approach to the treatment of multiple sclerosis. Nature 502, 327–332 (2013). 24107995 10.1038/nature12647 PMC4431622

[b25] Hammond T. R. *et al.* Astrocyte-derived endothelin-1 inhibits remyelination through notch activation. Neuron 81, 588–602 (2014). 24507193 10.1016/j.neuron.2013.11.015 PMC3935216

[b26] Yuen T. J. *et al.* Identification of endothelin 2 as an inflammatory factor that promotes central nervous system remyelination. Brain 136, 1035–1047 (2013). 23518706 10.1093/brain/awt024 PMC3613712

[b27] Golan N. *et al.* Identification of Tmem10/Opalin as an oligodendrocyte enriched gene using expression profiling combined with genetic cell ablation. Glia 56, 1176–1186 (2008). 18571792 10.1002/glia.20688 PMC2830273

[b28] Brockschnieder D., Sabanay H., Riethmacher D. & Peles E. Ermin, a myelinating oligodendrocyte-specific protein that regulates cell morphology. J. Neurosci. 26, 757–762 (2006). 16421295 10.1523/JNEUROSCI.4317-05.2006 PMC6675369

[b29] Marazziti D., Gallo A., Golini E., Matteoni R. & Tocchini-Valentini G. P. Molecular cloning and chromosomal localization of the mouse Gpr37 gene encoding an orphan G-protein-coupled peptide receptor expressed in brain and testis. Genomics 53, 315–324 (1998). 9799598 10.1006/geno.1998.5433

[b30] Zhang Y. *et al.* An RNA-sequencing transcriptome and splicing database of glia, neurons, and vascular cells of the cerebral cortex. J. Neurosci. 34, 11929–11947 (2014). 25186741 10.1523/JNEUROSCI.1860-14.2014 PMC4152602

[b31] Imai Y. *et al.* An unfolded putative transmembrane polypeptide, which can lead to endoplasmic reticulum stress, is a substrate of parkin. Cell 105, 891–902 (2001). 11439185 10.1016/s0092-8674(01)00407-x

[b32] Roesch K. *et al.* The transcriptome of retinal Muller glial cells. J. Comp. Neurol. 509, 225–238 (2008). 18465787 10.1002/cne.21730 PMC2665263

[b33] Zhang Y. *et al.* Purification and characterization of progenitor and mature human astrocytes reveals transcriptional and functional differences with mouse. Neuron 89, 37–53 (2016). 26687838 10.1016/j.neuron.2015.11.013 PMC4707064

[b34] Barateiro A. & Fernandes A. Temporal oligodendrocyte lineage progression: *in vitro* models of proliferation, differentiation and myelination. Biochim. Biophys. Acta 1843, 1917–1929 (2014). 24768715 10.1016/j.bbamcr.2014.04.018

[b35] Eisenbach M. *et al.* Differential clustering of Caspr by oligodendrocytes and Schwann cells. J. Neurosci. Res. 87, 3492–3501 (2009). 19565653 10.1002/jnr.22157

[b36] Pedraza L., Huang J. K. & Colman D. Disposition of axonal caspr with respect to glial cell membranes: implications for the process of myelination. J. Neurosci. Res. 87, 3480–3491 (2009). 19170162 10.1002/jnr.22004

[b37] Hirrlinger P. G. *et al.* Expression of reef coral fluorescent proteins in the central nervous system of transgenic mice. Mol. Cell. Neurosci. 30, 291–303 (2005). 16169246 10.1016/j.mcn.2005.08.011

[b38] Ishii A., Fyffe-Maricich S. L., Furusho M., Miller R. H. & Bansal R. ERK1/ERK2 MAPK signaling is required to increase myelin thickness independent of oligodendrocyte differentiation and initiation of myelination. J. Neurosci. 32, 8855–8864 (2012). 22745486 10.1523/JNEUROSCI.0137-12.2012 PMC3521511

[b39] Keshet Y. & Seger R. The MAP kinase signaling cascades: a system of hundreds of components regulates a diverse array of physiological functions. Methods Mol. Biol. 661, 3–38 (2010). 20811974 10.1007/978-1-60761-795-2_1

[b40] Plotnikov A. *et al.* The nuclear translocation of ERK1/2 as an anticancer target. Nat. Commun. 6, 6685 (2015). 25819065 10.1038/ncomms7685

[b41] Sala E. *et al.* BRAF silencing by short hairpin RNA or chemical blockade by PLX4032 leads to different responses in melanoma and thyroid carcinoma cells. Mol. Cancer. Res. 6, 751–759 (2008). 18458053 10.1158/1541-7786.MCR-07-2001

[b42] Galabova-Kovacs G. *et al.* Essential role of B-Raf in oligodendrocyte maturation and myelination during postnatal central nervous system development. J. Cell Biol. 180, 947–955 (2008). 18332218 10.1083/jcb.200709069 PMC2265404

[b43] Meyer R. C., Giddens M. M., Schaefer S. A. & Hall R. A. GPR37 and GPR37L1 are receptors for the neuroprotective and glioprotective factors prosaptide and prosaposin. Proc. Natl Acad. Sci. USA 110, 9529–9534 (2013). 23690594 10.1073/pnas.1219004110 PMC3677493

[b44] Emery B. & Lu Q. R. Transcriptional and epigenetic regulation of oligodendrocyte development and myelination in the central nervous system. Cold Spring Harb. Perspect. Biol. 7, a020461 (2015). 26134004 10.1101/cshperspect.a020461 PMC4563712

[b45] Hernandez M. & Casaccia P. Interplay between transcriptional control and chromatin regulation in the oligodendrocyte lineage. Glia 63, 1357–1375 (2015). 25970296 10.1002/glia.22818 PMC4470782

[b46] Bujalka H. *et al.* MYRF is a membrane-associated transcription factor that autoproteolytically cleaves to directly activate myelin genes. PLoS Biol. 11, e1001625 (2013). 23966833 10.1371/journal.pbio.1001625 PMC3742440

[b47] Emery B. *et al.* Myelin gene regulatory factor is a critical transcriptional regulator required for CNS myelination. Cell 138, 172–185 (2009). 19596243 10.1016/j.cell.2009.04.031 PMC2757090

[b48] Koenning M. *et al.* Myelin gene regulatory factor is required for maintenance of myelin and mature oligodendrocyte identity in the adult CNS. J. Neurosci. 32, 12528–12542 (2012). 22956843 10.1523/JNEUROSCI.1069-12.2012 PMC3752083

[b49] Liu A. *et al.* A molecular insight of Hes5-dependent inhibition of myelin gene expression: old partners and new players. EMBO J. 25, 4833–4842 (2006). 17006542 10.1038/sj.emboj.7601352 PMC1618116

[b50] Hornig J. *et al.* The transcription factors Sox10 and Myrf define an essential regulatory network module in differentiating oligodendrocytes. PLoS Genet. 9, e1003907 (2013). 24204311 10.1371/journal.pgen.1003907 PMC3814293

[b51] Brinkmann B. G. *et al.* Neuregulin-1/ErbB signaling serves distinct functions in myelination of the peripheral and central nervous system. Neuron 59, 581–595 (2008). 18760695 10.1016/j.neuron.2008.06.028 PMC2628490

[b52] Ishii A., Furusho M. & Bansal R. Sustained activation of ERK1/2 MAPK in oligodendrocytes and schwann cells enhances myelin growth and stimulates oligodendrocyte progenitor expansion. J. Neurosci. 33, 175–186 (2013). 23283332 10.1523/JNEUROSCI.4403-12.2013 PMC3711773

[b53] Hennen S. *et al.* Decoding signaling and function of the orphan G protein-coupled receptor GPR17 with a small-molecule agonist. Sci. Signal. 6, ra93 (2013). 24150254 10.1126/scisignal.2004350 PMC4114018

[b54] Boda E. *et al.* The GPR17 receptor in NG2 expressing cells: focus on *in vivo* cell maturation and participation in acute trauma and chronic damage. Glia 59, 1958–1973 (2011). 21956849 10.1002/glia.21237

[b55] Harlow D. E. & Macklin W. B. Inhibitors of myelination: ECM changes, CSPGs and PTPs. Exp. Neurol. 251, 39–46 (2014). 24200549 10.1016/j.expneurol.2013.10.017 PMC4060786

[b56] Mei F., Christin Chong S. Y. & Chan J. R. Myelin-based inhibitors of oligodendrocyte myelination: clues from axonal growth and regeneration. Neurosci. Bull. 29, 177–188 (2013). 23516141 10.1007/s12264-013-1319-x PMC5190510

[b57] Rosenberg S. S., Kelland E. E., Tokar E., De la Torre A. R. & Chan J. R. The geometric and spatial constraints of the microenvironment induce oligodendrocyte differentiation. Proc. Natl Acad. Sci. USA 105, 14662–14667 (2008). 18787118 10.1073/pnas.0805640105 PMC2567234

[b58] Lee S. *et al.* A culture system to study oligodendrocyte myelination processes using engineered nanofibers. Nat. Methods 9, 917–922 (2012). 22796663 10.1038/nmeth.2105 PMC3433633

[b59] Czopka T., Ffrench-Constant C. & Lyons D. A. Individual oligodendrocytes have only a few hours in which to generate new myelin sheaths *in vivo*. Dev. Cell 25, 599–609 (2013). 23806617 10.1016/j.devcel.2013.05.013 PMC4013507

[b60] Watkins T. A., Emery B., Mulinyawe S. & Barres B. A. Distinct stages of myelination regulated by gamma-secretase and astrocytes in a rapidly myelinating CNS coculture system. Neuron 60, 555–569 (2008). 19038214 10.1016/j.neuron.2008.09.011 PMC2650711

[b61] Back S. A., Luo N. L., Borenstein N. S., Volpe J. J. & Kinney H. C. Arrested oligodendrocyte lineage progression during human cerebral white matter development: dissociation between the timing of progenitor differentiation and myelinogenesis. J. Neuropathol. Exp. Neurol. 61, 197–211 (2002). 11853021 10.1093/jnen/61.2.197

[b62] Meyer R. C., Giddens M. M., Coleman B. M. & Hall R. A. The protective role of prosaposin and its receptors in the nervous system. Brain. Res. 1585, 1–12 (2014). 25130661 10.1016/j.brainres.2014.08.022 PMC4529117

[b63] Sheean M. E. *et al.* Activation of MAPK overrides the termination of myelin growth and replaces Nrg1/ErbB3 signals during Schwann cell development and myelination. Genes Dev. 28, 290–303 (2014). 24493648 10.1101/gad.230045.113 PMC3923970

[b64] Afshari F. S., Chu A. K. & Sato-Bigbee C. Effect of cyclic AMP on the expression of myelin basic protein species and myelin proteolipid protein in committed oligodendrocytes: differential involvement of the transcription factor CREB. J. Neurosci. Res. 66, 37–45 (2001). 11599000 10.1002/jnr.1195

[b65] Sun X., Liu Y., Liu B., Xiao Z. & Zhang L. Rolipram promotes remyelination possibly via MEK-ERK signal pathway in cuprizone-induced demyelination mouse. Exp. Neurol. 237, 304–311 (2012). 22836144 10.1016/j.expneurol.2012.07.011

[b66] Syed Y. A. *et al.* Inhibition of phosphodiesterase-4 promotes oligodendrocyte precursor cell differentiation and enhances CNS remyelination. EMBO Mol. Med. 5, 1918–1934 (2013). 24293318 10.1002/emmm.201303123 PMC3914530

[b67] Ishii A., Furusho M., Dupree J. L. & Bansal R. Role of ERK1/2 MAPK signaling in the maintenance of myelin and axonal integrity in the adult CNS. J. Neurosci. 34, 16031–16045 (2014). 25429144 10.1523/JNEUROSCI.3360-14.2014 PMC4244469

[b68] Poliak S. *et al.* Juxtaparanodal clustering of Shaker-like K+ channels in myelinated axons depends on Caspr2 and TAG-1. J. Cell. Biol. 162, 1149–1160 (2003). 12963709 10.1083/jcb.200305018 PMC2172860

[b69] Eshed Y. *et al.* Gliomedin mediates Schwann cell-axon interaction and the molecular assembly of the nodes of Ranvier. Neuron 47, 215–229 (2005). 16039564 10.1016/j.neuron.2005.06.026

