# Supplementary material for: G protein-coupled receptor 37 is a negative regulator of oligodendrocyte differentiation and myelination
Source: Nat Commun. 2016 Mar 10;7:10884. doi: 10.1038/ncomms10884 (PMC4792952; doi:10.1038/ncomms10884)
Supplement: Supplementary Information — Supplementary Figures 1-10 [file ncomms10884-s1.pdf]

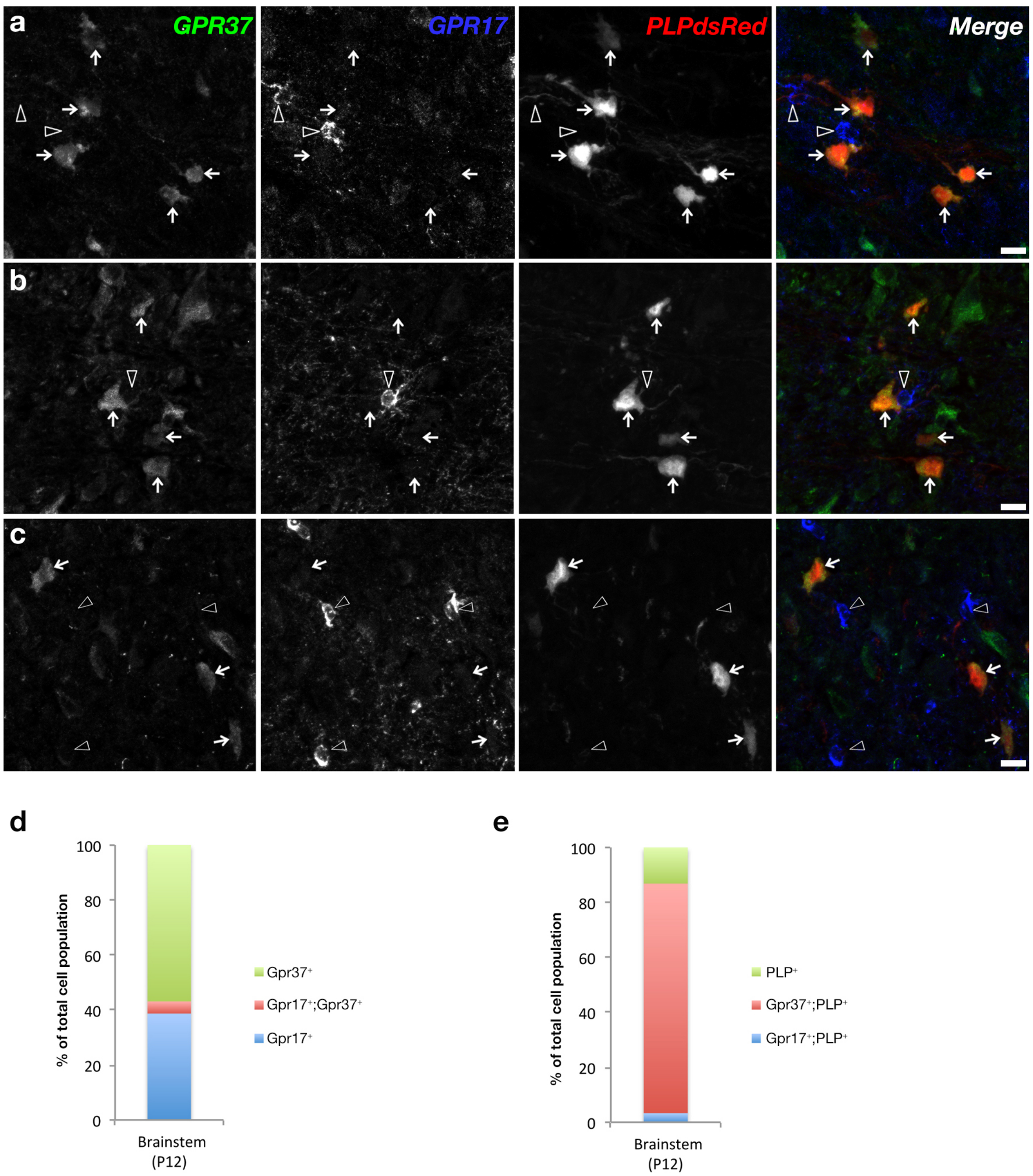

Supplementary Figure 1. Expression of GPR37 and GPR17 is mutually exclusive. a-c. Immunolabeling of P12 *PlpRed* mouse brainstems with an antibody to GPR37 and GPR17. Three representative pictures are shown. Arrows indicate cells expressing both GPR37 and PLPdsRed, while open triangles indicate cells expressing GPR17. Scale bars, 10 µm. **d.** Percentage of GPR17- and GPR37-positive cells among the total cell population expressing either GPR17 or GPR37 (n=204 cells from three different mouse brains). Only 4% of the cells were expressing both GPCRs. **e.** Percentage of cells expressing GPR17 or GPR37 among the total population of PLPdsRed-positive cells (n=61 cells from three different mouse brains).

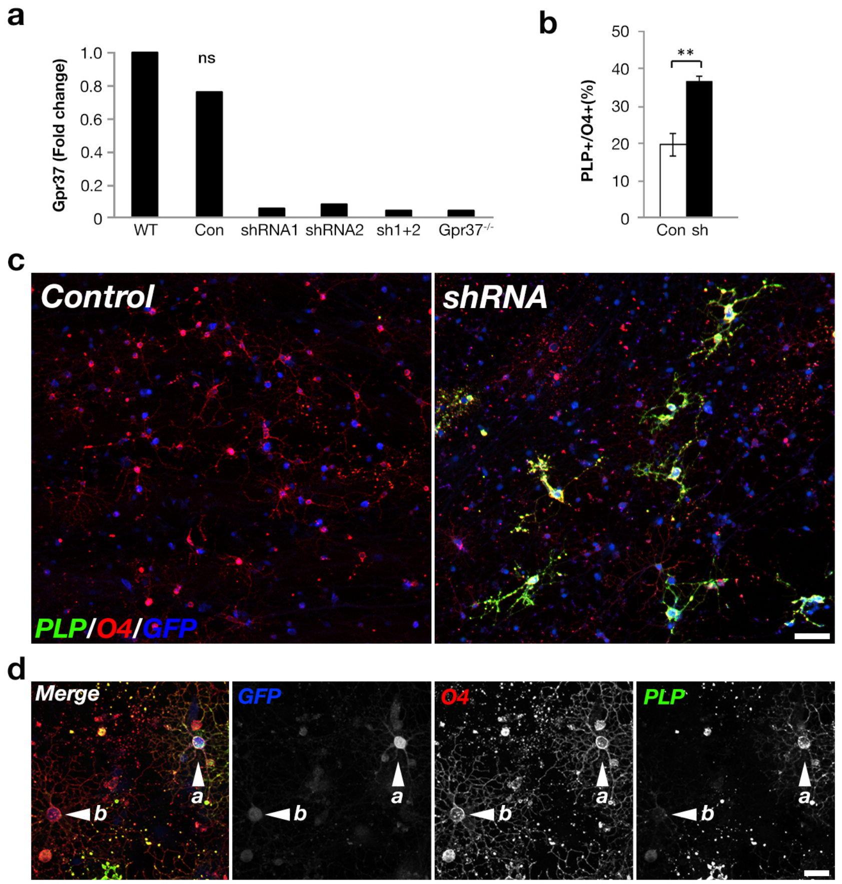

Supplementary Figure 2. shRNA knockdown of GPR37 enhances oligodendrocyte differentiation. **a.** qPCR analysis of OPC cultures at DIV5. Expression of GPR37 in OPC cultures infected with retroviruses containing pSuper-shRNA vectors (shRNA1 or shRNA2 or combined 'sh1+2'), compared to wild type (WT), vector alone (Con) or OPCs isolated from *Gpr37*$^{-/-}$ mice. ns, not significant. **b.** Percentage of PLP positive oligodendrocytes among the total population of O4 positive cells in empty vectors (Con)- or combined shRNA vectors (sh)-infected OPCs co-cultured with DRG neurons. The number of cells expressing PLP at DIV3 is significantly higher in GPR37 knockdown oligodendrocytes (***P*<0.01, n=3 cultures, *t*-test). Bars represent mean ± SEM. **c-d.** Immunostaining of infected OPCs co-cultured with DRG neurons at DIV3, using antibodies to PLP, O4 and GFP (GFP was used to mark infected cells). **d.** Higher magnification of shRNA-infected cocultures showing the increase in PLP expression in a cell expressing high (a), but not low (b) levels of GFP. Scale bar, 50 µm (c), 20 µm (d).

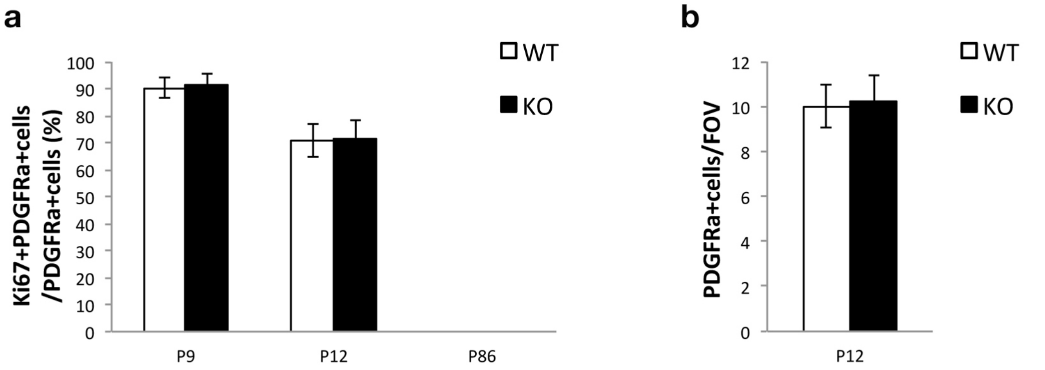

Supplementary Figure 3. Absence of Gpr37 does not affect OPC proliferation. a. Longitudinal sections of optic nerves isolated from wild type (WT) and *Gpr37*$^{-/-}$ (KO) mice at P9, P12, and P86 were immunolabeled using Ki67 and PDGFRα antibodies. The graph shows the percentage of proliferating OPCs (Ki67/PDGFRα) at the different ages. The following number of sections from 2 mice of each genotype was used for analyses of each age (P9: WT=9, KO=8; P12: WT=10, KO=10; P86: WT=11, KO=12). **b.** Number of PDGFRα cells present in P12 optic nerves isolated from *Gpr37*$^{-/-}$ mice and their littermate controls per field of view (FOV). Ten images (magnification 63x) from two mice per genotype were analyzed. Bars represent mean ± SEM.

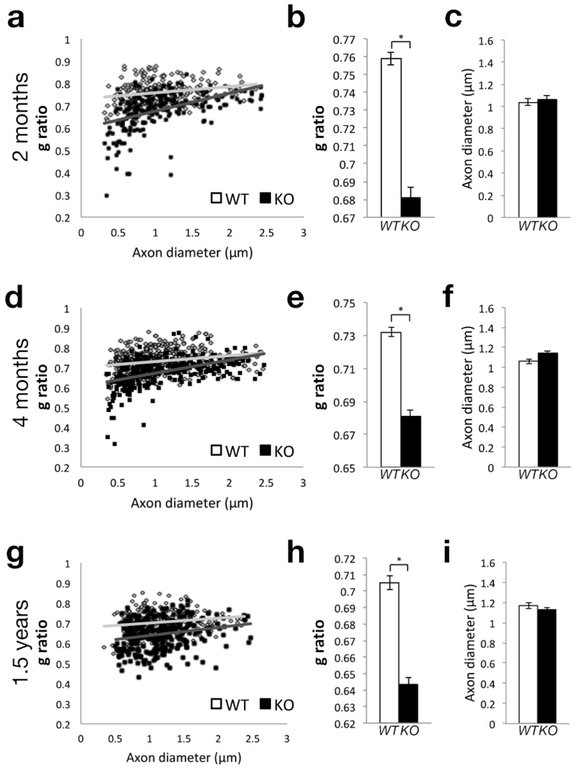

Supplementary Figure 4. The absence of Gpr37 resulted in thicker myelin in the spinal cord. Quantification of myelin thickness was done by analyzing electron microscope images of spinal cords isolated from wild type (WT) and *Gpr37*$^{-/-}$ (KO) mice at the age of 2 months (**a-c**), 4 months (**d-f**) and 1.5 years (**g-i**). **a,d,g.** g-ratio is presented as a function of the corresponding axon diameter. Total of 210 axons (WT) and 223 axons (KO) from 2 mice (for 2 month), 438 axons (WT) and 334 axons (KO) from 4 WT mice and 3 KO mice (for 4 month), 206 axons (WT) and 315 axons (KO) from 2 mice (for 1.5 year) of each genotype were examined for the analysis. **b,e,h.** The averaged g-ratio is significantly lower in *GPR37*$^{-/-}$ than *WT* (*$P<0.001$, *t*-test). **c,f,i.** Averaged axonal diameter is not significantly changed in spinal cord of *Gpr37*$^{-/-}$ and *WT.* Bars represent mean ± SEM.

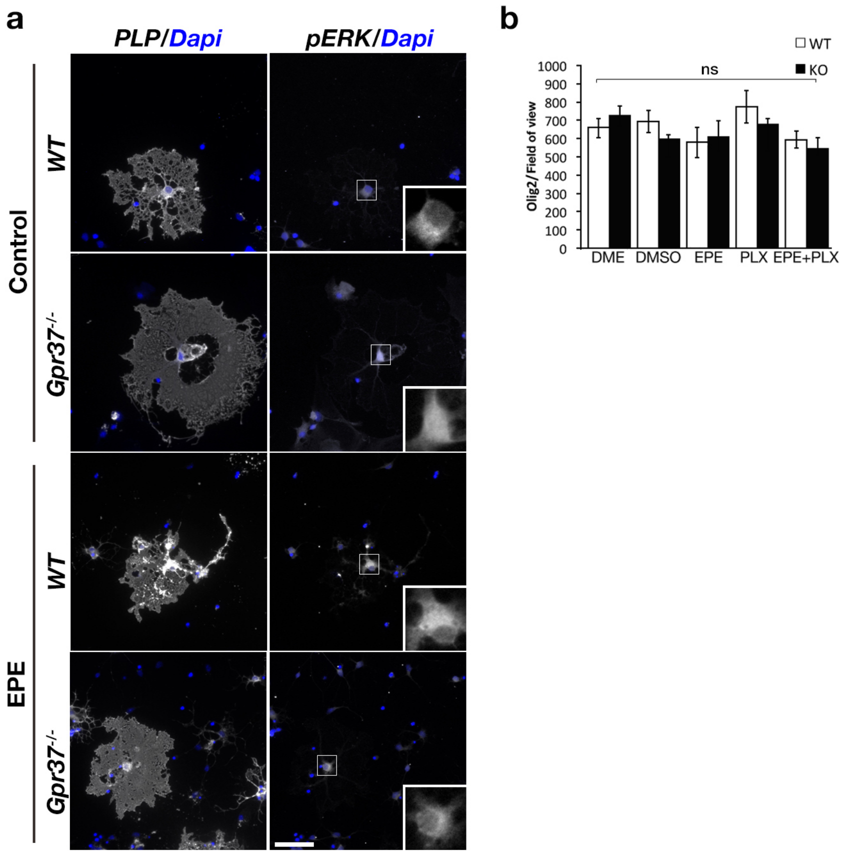

Supplementary Figure 5. EPE blocks the nuclear translocation of pERK in Gpr37 null oligodendrocytes. a. Cultured OPCs isolated from *WT* and *Gpr37*$^{-/-}$ mice were grown for 3 days (DIV2-DIV5) in the presence or absence (control) of EPE, followed by immunolabeling with antibodies to pERK and PLP. The nuclei were labeled with Dapi. PLP and pERK immunoreactivity are shown in separate images along with the Dapi signal. Higher magnification of the nucleus is shown in the insets in each panel. EPE prevented the nuclear translocation of pERK seen in *Gpr37*$^{-/-}$ oligodendrocytes. Scale bar, 50 µm. **b.** ERK inhibitors do not affect the number of oligodendrocyte co-cultured with DRG neurons. Cultures were grown in the absence (DME), or presence of EPE, PLX4032, or DMSO as control for 6 days, and then fixed at DIV7 and labeled with antibodies to Olig2. The number of positive cells per field of view is shown in the graph. Bars represent mean ± SEM (n=3 different cultures per each genotype; 10 fields of view were analyzed for each culture).

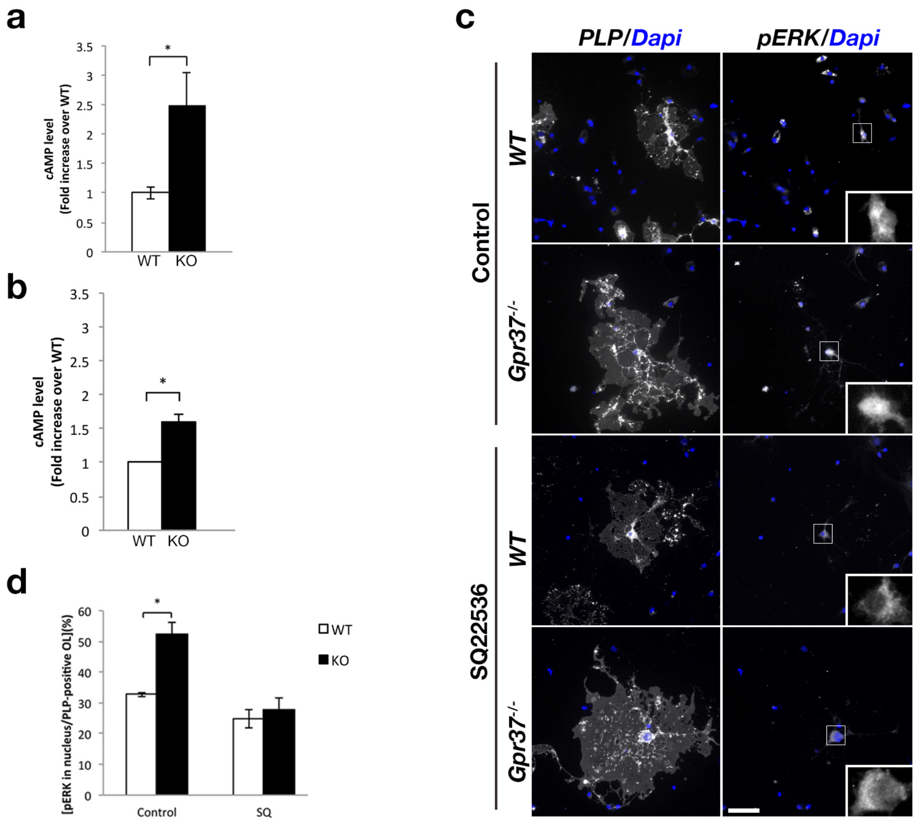

Supplementary Figure 6. Involvement of cAMP in Gpr37 signaling in oligodendrocytes. a-b. Absence of Gpr37 results in increased levels of cAMP in mice brain (a) and cultured OPCs (b). Intracellular cAMP was extracted from P70 mice brains (* P<0.05, *t*-test; n=6 WT and n=7 KO mice), or from DIV5 cultured OPCs (* P<0.01, *t*-test; n=3 different OPC primary cultures of each genotype) and detected by ELISA. **c.** Adenylate cyclase inhibition prevents the nuclear translocation of pERK in *Gpr37*$^{-/-}$ OPCs. Cultured wild type (WT) and *Gpr37*$^{-/-}$ OPCs (DIV7) were left untreated (control) or treated with the adenylyl cyclase inhibitor SQ22536 (0.1mM) for 2 hours in their growth medium. Cells were then fixed and labeled with Dapi to mark their nuclei, and with antibodies to PLP and pERK. PLP and pERK immunoreactivity are shown in separate panels along with the Dapi signal. Insets show higher magnification of the boxed area in each panel. Scale bar: 50 µm. **d.** Quantification of the immunolabeling, showing that the nuclear localization of pERK in *Gpr37*$^{-/-}$ oligodendrocytes was reduced to equivalent levels of wild type cells after the treatment. Bars show mean ± SEM (* P<0.01, *t*-test; n=3 different primary cultures for each genotype; 50 PLP-positive cells were counted per each culture).

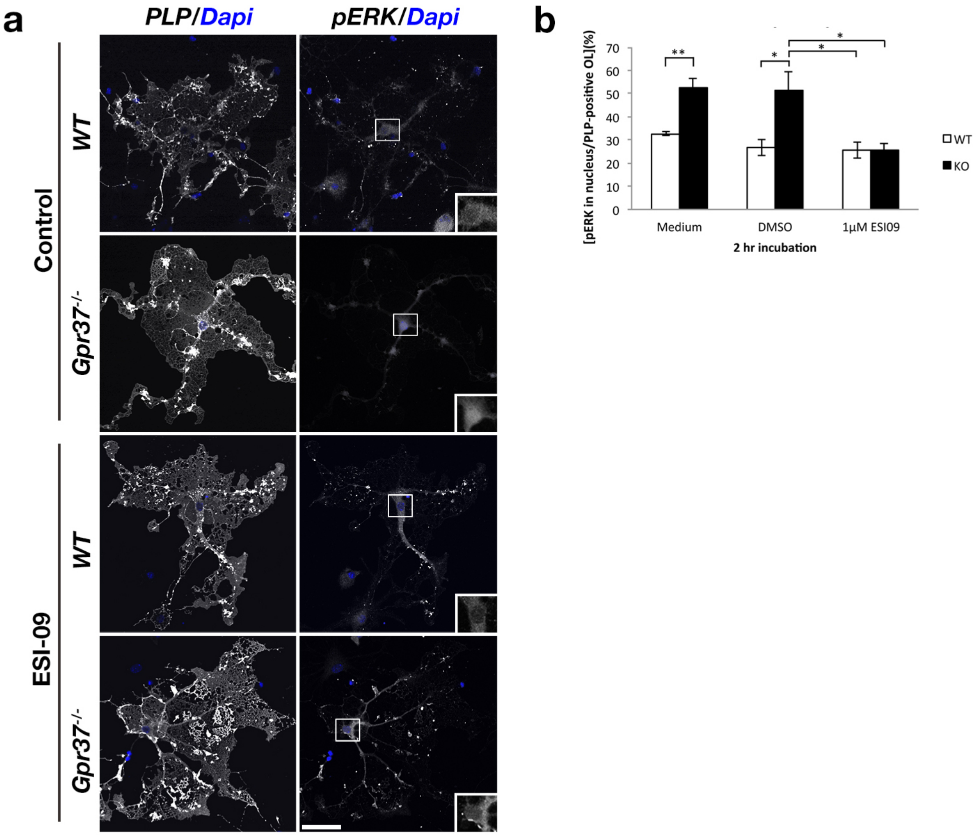

Supplementary Figure 7. Inhibition of Epac activity attenuates nuclear translocation of ERK1/2 in oligodendrocytes lacking GPR37. a. Cultured wild type (WT) and *Gpr37*$^{-/-}$ OPCs (DIV7) were treated with DMSO (Control) or with the Epac inhibitor ESI-09 (1μM) for 2 hours in their growth medium. Cells were then fixed and labeled with Dapi to mark their nuclei, and with antibodies to PLP and phosphorylated ERK (pERK). PLP and pERK immunoreactivity are shown in separate panels along with the Dapi signal. Insets show higher magnification of the boxed area in each panel. Scale bar: 40 μm. **b.** Quantification of the immunolabeling, showing that the nuclear localization of pERK in GPR37$^{-/-}$ oligodendrocytes was reduced to equivalent level of wild type cells after the treatment. Bars show mean ± SEM (* *P*<0.05, ** *P*<0.01, *t*-test; n=3 different primary cultures for each genotype; 100 PLP-positive cells were counted per each culture).

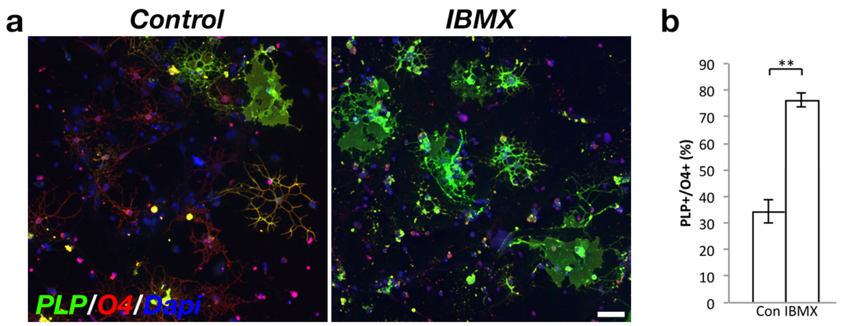

Supplementary Figure 8. cAMP enhances oligodendrocyte differentiation. a. Immunolabeling of wild type OPC cultures grown for four days in the absence (Control) or presence of 1mM 3-isobutyl-1-methylxanthine (IBMX) using antibodies to PLP and O4. Dapi was used to label the nuclei. Scale bar, 50 µm. **b.** Percentage of PLP-positive oligodendrocytes among the total O4-positive cells (***P*<0.01, *t*-test, n=3 cultures). Bars represent mean ± SEM.

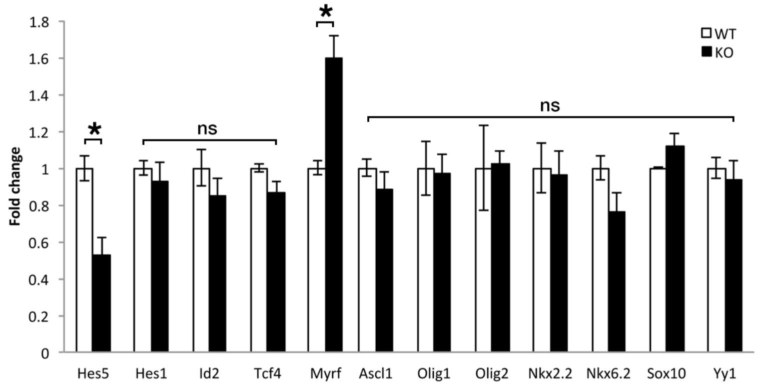

Supplementary Figure 9. Absence of GPR37 results in an increase in Myrf and a decrease in Hes5 expression. qRT-PCR analysis of P4 brainstems isolated from wild type (WT) and *GPR37*$^{-/-}$ (KO) mice. Gene expression levels of the other indicated transcription factors do not show a significant change. Bars represent mean ± SEM, WT=3 mice, KO=6 mice; **P*=0.01, *t*-test.

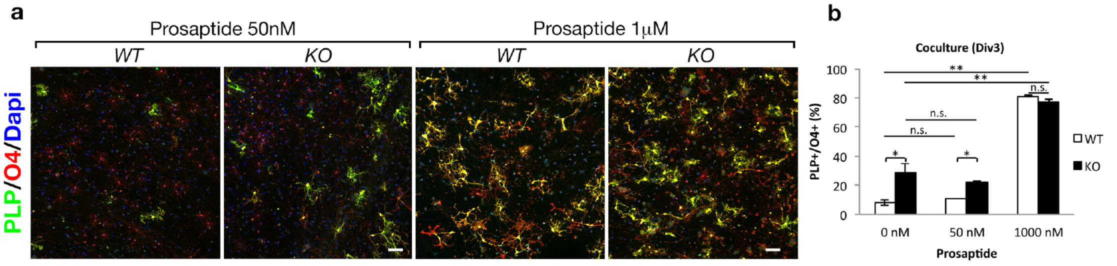

Supplementary Figure 10. Effect of prosaptide on oligodendrocyte differentiation. a. OPC/DRG neuron cocultures were grown with the indicated concentration of prosaptide (ANASPEC) for three days. Cells were then labeled with antibodies to PLP and O4. Dapi was used to stain the nuclei. Scale bar, 100 µm. **b.** Percentage of PLP-positive oligodendrocytes among the total cell populations expressing O4 is shown. **P*<0.05, ***P*<0.01, *t*-test, n=3 cultures per each genotype at each concentration. Bars represent mean ± SEM. n.s., not significant.

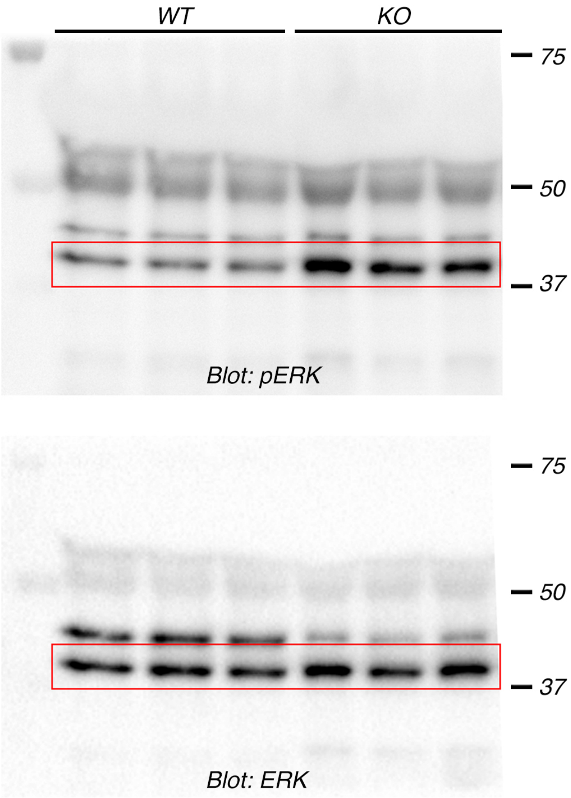

Supplementary Figure 11. Original Western blots of OPC cultures using antibodies to phosphorylated (pERK) and general ERK (ERK). Three different OPC cultures from wild type (WT) and *Gpr37*$^{-/-}$ (KO) mice are shown along with the location of molecular mass markers in kDa. The framed area is shown in Figure 5c.
